# Molecular epidemiology of dengue viruses in three provinces of Lao PDR, 2006-2010

**DOI:** 10.1371/journal.pntd.0006203

**Published:** 2018-01-29

**Authors:** Josée Castonguay-Vanier, Raphaëlle Klitting, Onanong Sengvilaipaseuth, Géraldine Piorkowski, Cécile Baronti, Bountoy Sibounheuang, Manivanh Vongsouvath, Anisone Chanthongthip, Soulignasack Thongpaseuth, Mayfong Mayxay, Koukeo Phommasone, Phouvieng Douangdala, Saythong Inthalath, Phouthalavanh Souvannasing, Paul N. Newton, Xavier de Lamballerie, Audrey Dubot-Pérès

**Affiliations:** 1 Lao-Oxford-Mahosot Hospital-Wellcome Trust Research Unit (LOMWRU), Microbiology Laboratory, Mahosot Hospital, Vientiane, Lao PDR; 2 Centre for Tropical Medicine & Global Health, Nuffield Department of Clinical Medicine, University of Oxford, Churchill Hospital, Oxford, United Kingdom; 3 UMR "Unité des Virus Emergents" (UVE: Aix-Marseille Univ–IRD 190 –Inserm 1207 –IHU Méditerranée Infection), Marseille, France; 4 Faculty of Postgraduate Studies, University of Health Sciences, Vientiane, Lao PDR; 5 Luang Namtha Provincial Hospital, Luang Namtha, Luang Namtha Province, Lao PDR; 6 Salavan Provincial Hospital, Sekong, Salavan Province, Lao PDR; University of Texas Medical Branch, UNITED STATES

## Abstract

Few data on dengue epidemiology are available for Lao PDR. Here, we provide information on the complexity of dengue epidemiology in the country, demonstrating dynamic circulation that varies over space and time, according to serotype. We recruited 1,912 consenting patients presenting with WHO dengue criteria at Mahosot Hospital, Vientiane (central Laos), between 2006 and 2010. Between 2008 and 2010, 1,413 patients with undifferentiated fever were also recruited at Luang Namtha (LNT) Provincial Hospital (northern Laos) and 555 at Salavan (SV) Provincial Hospital (southern Laos). We report significant variations in *Dengue virus* (DENV) circulation between the three sites. Peaks of DENV infection were observed in the rainy seasons, although 11% of confirmed cases in the provinces and 4.6% in the capital were detected during the dry and cool seasons (between December and February). Four DENV serotypes were detected among the 867 RT-PCR positive patients: 76.9% DENV-1, 9.6% DENV-2, 7.7% DENV-4 and 5.3% DENV-3. DENV-1 was the predominant serotype throughout the study except in LNT in 2008 and 2009 when it was DENV-2. Before July 2009, DENV-2 was not detected in SV and only rarely detected in Vientiane. DENV-3 and DENV-4 were commonly detected in Vientiane, before 2008 for DENV-4 and after 2009 for DENV-3. The phylogenetic analyses of DENV envelope sequences suggest concurrent multiple introductions of new strains as well as active DENV circulation throughout Laos and with neighboring countries. It is therefore of great importance to develop and strengthen a year-round nation-wide surveillance network in order to collect data that would allow anticipation of public health issues caused by the occurrence of large dengue outbreaks.

## Author summary

Dengue is a mosquito-borne disease that can be caused by 4 viruses. It is a flu-like disease but can sometime be more severe and cause hemorrhage or death. An estimated 390 million people are infected every year, mainly in the Western Pacific and in South-East Asia. In Laos, where our study was conducted from 2006 to 2010, little was known on the circulation of the different dengue viruses. A total of 1,912 patients were recruited at Mahosot Hospital, Vientiane (central Laos), 1,413 patients at Luang Namtha Provincial Hospital (northern Laos) and 555 at Salavan Provincial Hospital (southern Laos). Although most *Dengue virus* infections were detected during rainy seasons (where mosquitoes are the most active) in all three provinces, some patients were also infected during the dryer months. All 4 dengue viruses were detected with different distributions: mostly type 1 in the capital and in the South, and type 1 and 2 in equal proportion in the north. Type 3 and 4 were not detected in Luang Namtha and rarely in Salavan. Comparison of *Dengue virus* sequences from Laos with sequences collected worldwide showed an active year-round circulation of dengue within Laos and with neighboring countries. It is hence of great importance to develop and strengthen a year-round nation-wide surveillance network in order to collect data that would allow anticipation of public health issues caused by the occurrence of large dengue outbreaks.

## Introduction

Dengue is an arboviral disease transmitted to humans by *Aedes* mosquitoes. Infections are caused by single-stranded positive-sense RNA *Dengue virus* (DENV) from the *Flavivirus* genus, *Flaviviridae* family. Any of the four virus serotypes (DENV-1 to DENV-4) can cause dengue fever, dengue hemorrhagic fever and dengue shock syndrome [1]; now regrouped under dengue with or without warning signs and severe dengue [2]. Infection by one of the 4 DENV serotypes confers lifelong immunity to that serotype only, as each is antigenically distinct [3]. Several factors such as prior immunity, viral load and infecting genotype or strain are believed to contribute to the severity of DENV infections [4,5]. The risk of severe dengue occurrence also has to be viewed through spatial and temporal distribution of concurrent or sequential circulation of DENV serotypes [6].

DENV is widespread in tropical and subtropical areas and is endemic in more than 100 countries [7]. There are an estimated 390 million DENV infections per year, with only 96 million being symptomatic [7]. According to WHO latest estimates, 500,000 people are requiring hospitalization every year, with a ~2.5% mortality [8]. Also worrying is that dengue’s apparent global burden has increased four-fold in the past 30 years [9]. Now, 3.9 billion people are considered at risk of contracting dengue, 70% of those live in the Western Pacific and in South-East Asia [8].

Lao PDR (Laos) is a low-middle income country of ~6.5 million people [10] bordered by China, Vietnam, Cambodia, Thailand and Myanmar. In Laos, DENV infection is a major cause of morbidity with a rising fatality rate [11], and approximately 3.9 million people are thought to be at risk of contracting a DENV infection [12]. Although hospitalized dengue cases have been reported since 1979 and recorded in a national database since 2008 [13,14], very few have been laboratory-confirmed [11]. Because only a limited number of studies have been conducted so far [12,14–22], little is known about the epidemiology of dengue or the DENV serotypes circulating within the country.

Here, we present DENV molecular epidemiological data from patients at 3 different hospitals in Laos: the provincial hospital of Luang Namtha in the north and the provincial hospital of Salavan in the south for 2008 to 2010, and Mahosot Hospital in the capital city of Vientiane (central Laos) for 2006 to 2010.

## Materials and methods

### Patients and study sites

#### Vientiane capital

**Fig 1:**
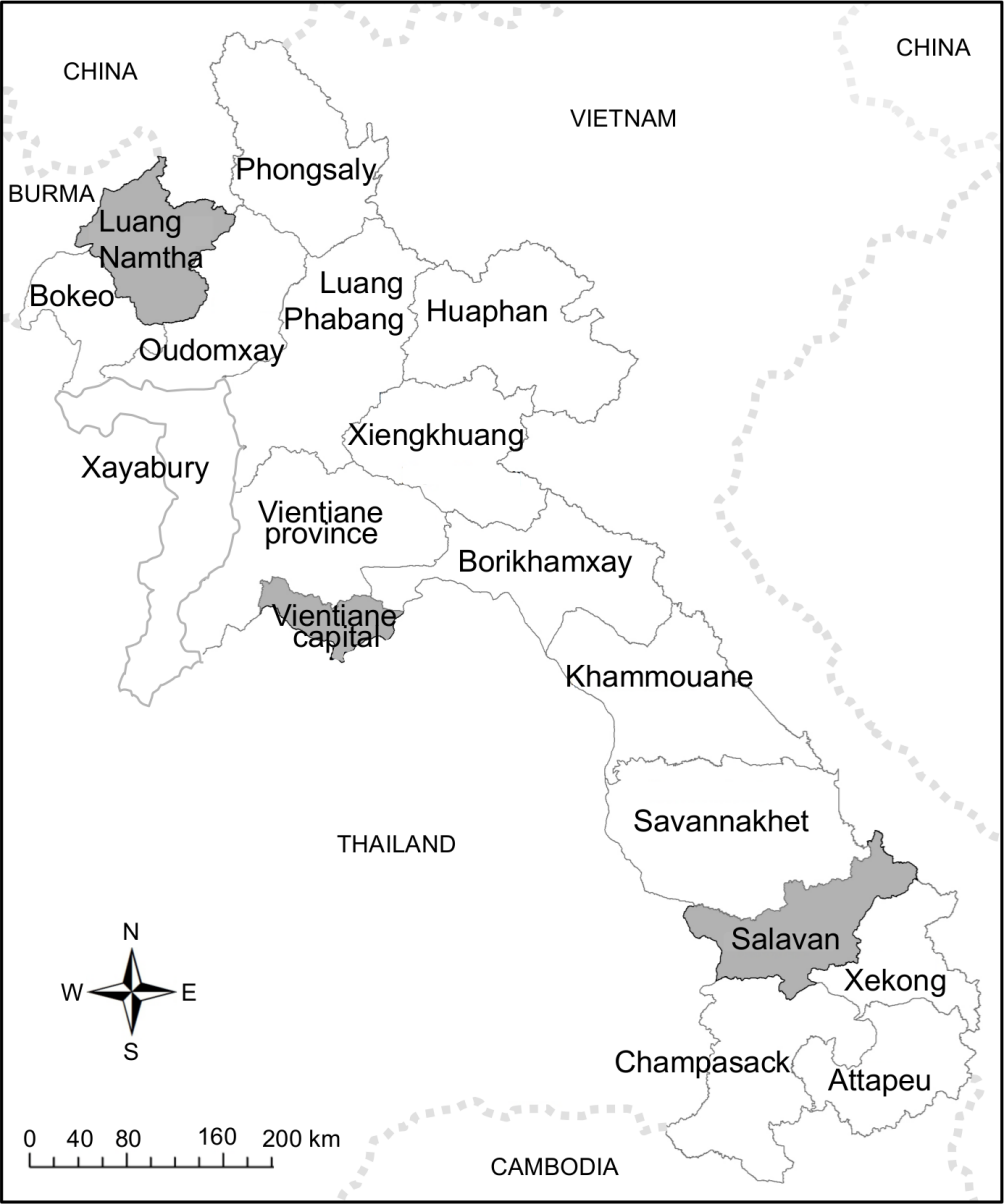
Map of the Lao PDR with Vientiane, Luang Namtha and Salavan study sites. Fig 1 is adapted from [23].

The study was conducted at Mahosot Hospital in the capital city of Vientiane (central Laos) from January 2006 to December 2010. Mahosot is a 365-bed primary-tertiary hospital, (17° 58’ N 102° 36’ E, at 174m above mean sea level), with approximately 2,000 admissions/month. The capital is bordered by the Mekong River with Thailand on the other bank (Fig 1) and has a population of ~820,000 (Population and Housing Census 2015, Lao Statistics Bureau). Hospital-admitted patients were included in this study if they gave written informed consent and if their responsible physician suspected dengue based on the 1997 WHO guidelines and requested laboratory tests.

#### Luang Namtha province

Luang Namtha (LNT) province, a mountainous region, is located in the far north-west of the country, bordering China and Myanmar (Fig 1). Its provincial hospital (21° 00’ N 101° 24’ E, at 570m above mean sea level), has 60 beds and serves a population of ~145,000 people. Patients were recruited from May 2008 until December 2010 [19]. In- and outpatients aged 5–49 years were enrolled if they had fever for less than 8 days without obvious causes, had admission tympanic temperature of > = 38°C and gave written informed consent.

#### Salavan province

Salavan (SV) Province, in southern Laos is bordered by Vietnam to the east and Thailand to the west (Fig 1). The provincial hospital (15° 43’ N 106° 25’ E, at 184m above mean sea level) has 70 beds and serves a population of ~332,000. Recruitment ranged from September 2008 until December 2010 with the same recruitment criteria as in LNT [19].

### Ethics statement

Written informed consent was obtained from all recruited patients or responsible guardians. Ethics approval was obtained from the Lao National Ethics Committee for Health Research and the Oxford Tropical Research Ethics Committee.

### Clinical samples

Venous blood was collected on admission from all patients and during convalescence when possible. Venous blood non-anticoagulated specimens were transported to Vientiane at ambient temperature within 48h such as described [19]. Serum samples were centrifuged at Mahosot Hospital upon reception and then stored at -80°C until use.

### ELISA assay

The following Panbio Ltd. (now Alere Inc, Waltham, Massachusetts, USA) ELISA kits were used to investigate DENV infections according to manufacturer’s instructions and as described [19,23]: Dengue Early ELISA (Cat no. E-DEN01P), Japanese encephalitis-dengue IgM Combo ELISA (Cat no. E-JED01C) and Dengue IgG capture ELISA (Cat no. E-DEN02G).

### RNA extractions

All admission sera from SV and LNT patients and admission sera from dengue ELISA positive patients from Mahosot were extracted with the QIAamp Viral RNA Mini kit (Qiagen, AG, Hombrechtikon, Switzerland) according to manufacturer’s instructions. The starting volume was 140μL while the final elution volume was 80μL. Internal phage control was added to all samples in order to monitor the extraction process and to check for PCR inhibitors [24].

### Real-time RT-PCR assay

The detection of serotypes 1–4 DENV RNA was performed in a single step TaqMan real-time reverse transcription PCR with the SuperScript III Platinum One-Step qRT-PCR kit (ThermoFisher Scientific, Waltham, Massachusetts, USA). Primers and probes followed Leparc-Goffart *et al*. [25] and 5μL of RNA extract was used as a template in a 25μL reaction volume. Positive samples were further characterised by using serotype-specific primers and probes [25].

### Results interpretation

Results were classified according to the USA CDC’s definition [26]. Confirmed dengue patients were those either with positive RT-PCR, positive NS1 ELISA or when a negative admission serum was paired with a convalescent serum positive for anti-dengue IgM or IgG. Presumptive dengue patients were those with anti-dengue antibody detection alone and no seroconversion.

Patients with confirmed and/or presumptive dengue were classified as dengue patients.

### Virus isolation

Isolation of dengue viruses were performed in a biosafety level 3 laboratory at the Infectious Disease Centre in Mahosot Hospital, from dengue patient sera as described [23]. Following one passage in a 25cm^2^ flask, RNA was extracted from 140μL of cell culture supernatant with the QIAamp Viral RNA kit according to manufacturer’s instruction (Qiagen, AG, Hombrechtikon, Switzerland). DENV RT-PCR was then performed as described above.

### *Dengue virus* genome sequencing

Specific amplifications of DENV genomes were performed from RNA extracted from cell culture or, when culture was not available, from patient serum samples. Amplicons were then sequenced by next-generation sequencing using Ion Torrent Personal Genome Machine (ThermoFisher Scientific, Waltham, Massachusetts, USA) as described by Baronti *et al*. [27] in Marseille, France, at the Faculty of Medicine, Emerging Viruses Unit.

### Sequences analysis

#### Data sets

DENV sequences were extracted from a database of all *Flavivirus* genus complete coding DNA sequences (CDS) available on GenBank at the end of the year 2013. The DENV complete CDS data sets included 1,373 sequences for DENV-1, 998 sequences for DENV-2, 670 sequences for DENV-3 and 123 sequences for DENV-4. All envelope sequences available on the European Molecular Biology Laboratory (EMBL) Nucleotide Sequence Database in April 2014 were recovered in order to build envelope datasets for each serotype. Unsuitable sequences (length <1,000 bases, number of N>50) were removed. Final datasets for envelope sequences included 3,108 sequences for DENV-1, 3,067 sequences for DENV-2, 1,817 sequences for DENV-3, 859 sequences for DENV-4.

Sequences obtained for this study were aligned with those datasets using ClustalX 2.1 [28].

#### Recombination

Indication of molecular recombination was investigated for each alignment of complete CDS using the Recombination Detection Program (RDP) version 4 software [29]. RDP, GENCONV and MAXCHI methods were used for primary screening and BOOTSCAN and SISCAN methods were used to check for recombination signals [29–33]. For optimal recombination detection, the automask procedure was selected. Recombination events with an average p-value with RDP lower than E-10 were selected for downstream phylogenetic analyses. Neighbor-Joining trees using Mega 6.06 software with Kimura-2 model [34], bootstrap resampling with 500 replicates, were built from the alignment used for RDP analysis. Two trees were produced and compared for each recombination event: one using sequences located between the putative recombination breakpoint positions and a second one which excluded the putative recombinant region.

#### Phylogenetic analysis

The best maximum-likelihood (ML) method to be used for tree building was determined for each serotype alignment using the MEGA6 software. For each serotype, two trees were subsequently built with either the complete CDS or the envelope alignment using the best ML method previously determined (General Time Reversible Model with invariant sites and a gamma distribution of rates across sites for DENV-1 and DENV-4 alignments, General Time-Reversible Model with gamma distributed rates across sites for DENV-2 alignment and Tamura-Nei model with gamma distributed rates across sites for DENV-3 alignment) and bootstrap resampling with 500 replicates on RAXML8.0.0 software [35].

## Results

### Dengue patients

#### Vientiane capital

A total of 1,912 patients with suspected dengue were recruited in Vientiane between January 2006 and December 2010 (Table 1). Dengue was confirmed in 922 patients (48.2%) and a further 221 (11.6%) were classified as having a presumptive dengue infection.

**Table 1:** *Dengue virus* infection and serotypes detected per site and per year.

	Number of patients (percentage)					
	Total	2006	2007	2008	2009	2010
**Vientiane**						
Recruited patients	1,912	309	786	170	150	497
Dengue*	1,143 (59.8)	157 (50.8)	384 (48.9)	50 (29.4)	122 (81.3)	430 (86.5)
Confirmed dengue	922 (48.2)	111 (35.9)	276 (35.1)	34 (20.0)	117 (78.0)	384 (77.0)
Presumptive dengue	221 (11.6)	46 (14.9)	108 (13.7)	16 (9.4)	5 (3.3)	46 (9.3)
RT-PCR positive	724 (37.9)	63 (20.4)	200 (25.4)	21 (12.4)	83 (55.3)	357 (71.8)
DENV-1	560 (77.3)	37 (58.7)	159 (79.5)	18 (85.7)	69 (83.1)	277 (77.6)
DENV-2	55 (7.6)	0	2 (1.0)	1 (4.8)	14 (16.9)	38 (10.6)
DENV-3	44 (6.1)	0	2 (1.0)	1 (4.8)	0	41 (11.5)
DENV-4	62 (8.6)	25 (39.7)	37 (18.5)	0	0	0
Co infection [types]	1 [1&4] 2 [1&2]	1 [1&4]	0	1 [1&2]	0	1 [1&2]
**Luang Namtha**						
Recruited patients	1,413	NA	NA	383	447	583
Dengue*	223 (15.8)			54 (14.1)	67 (15.0)	102 (17.5)
Confirmed dengue	90 (6.4)			15 (3.9)	27 (6.0)	48 (8.2)
Presumptive dengue	133 (9.4)			39 (10.2)	40 (8.9)	54 (9.3)
RT-PCR positive	34 (2.4)			8 (2.1)	10 (2.2)	16 (2.7)
DENV-1	19 (55.9)			1 (12.5)	4 (40)	14 (87.5)
DENV-2	14 (41.2)			6 (75.0)	6 (60)	2 (12.5)
DENV-3	0			0	0	0
DENV-4	0			0	0	0
Co infection [types]	1 [1&2]			1 [1&2] (0.3)	0	0
**Salavan**						
Recruited patients	555	NA	NA	77	276	202
Dengue*	210 (37.8)			25 (32.5)	104 (37.7)	81 (40.1)
Confirmed dengue	147 (26.5)			15 (19.5)	75 (27.2)	57 (28.2)
Presumptive dengue	63 (11.4)			10 (13.0)	29 (10.5)	24 (11.9)
RT-PCR positive	109 (19.6)			12 (15.6)	61 (22.1)	36 (17.8)
DENV-1	88 (80.7)			12 (100)	50 (82.0)	26 (72.2)
DENV-2	14 (12.8)			0	4 (6.6)	10 (27.8)
DENV-3	2 (1.8)			0	2 (3.3)	0
DENV-4	5 (4.6)			0	5 (8.2)	0
Co infection [types]	0			0	0	0
**Total**						
Recruited patients	3,880	309	786	630	873	1282
Dengue*	1,576 (40.6)	157 (50.8)	384 (48.9)	129 (20.5)	293 (33.6)	613 (47.8)
Confirmed dengue	1,159 (29.9)	111 (35.9)	276 (35.1)	64 (10.2)	219 (25.1)	489 (38.1)
Presumptive dengue	417 (10.7)	46 (14.9)	108 (13.7)	65 (10.3)	74 (8.5)	124 (9.7)
RT-PCR positive	867 (22.3)	62 (20.1)	200 (25.4)	41 (6.5)	154 (17.6)	409 (31.9)
DENV-1	667 (76.9)	36 (58.1)	159 (79.5)	31 (75.6)	123 (79.9)	317 (77.5)
DENV-2	83 (9.6)	0	2 (1.0)	7 (17.1)	24 (15.6)	50 (12.2)
DENV-3	46 (5.3)	0	2 (1.0)	1 (2.4)	2 (1.3)	41 (10.0)
DENV-4	67 (7.7)	25 (40.3)	37 (18.5)	0	5 (3.2)	0
Co infection [types]	1 [1&4] 3 [1&2]	1 [1&4]	0	2 [1&2] (0.3)		1 [1&2]

Dengue*: patients with confirmed and/or presumptive dengue. Confirmed dengue: positive either by dengue RT-PCR, NS1 ELISA or anti-dengue IgM or IgG seroconversion. Presumptive dengue: antibody detection alone with no seroconversion. DENV: *Dengue virus*.

**Fig 2:**
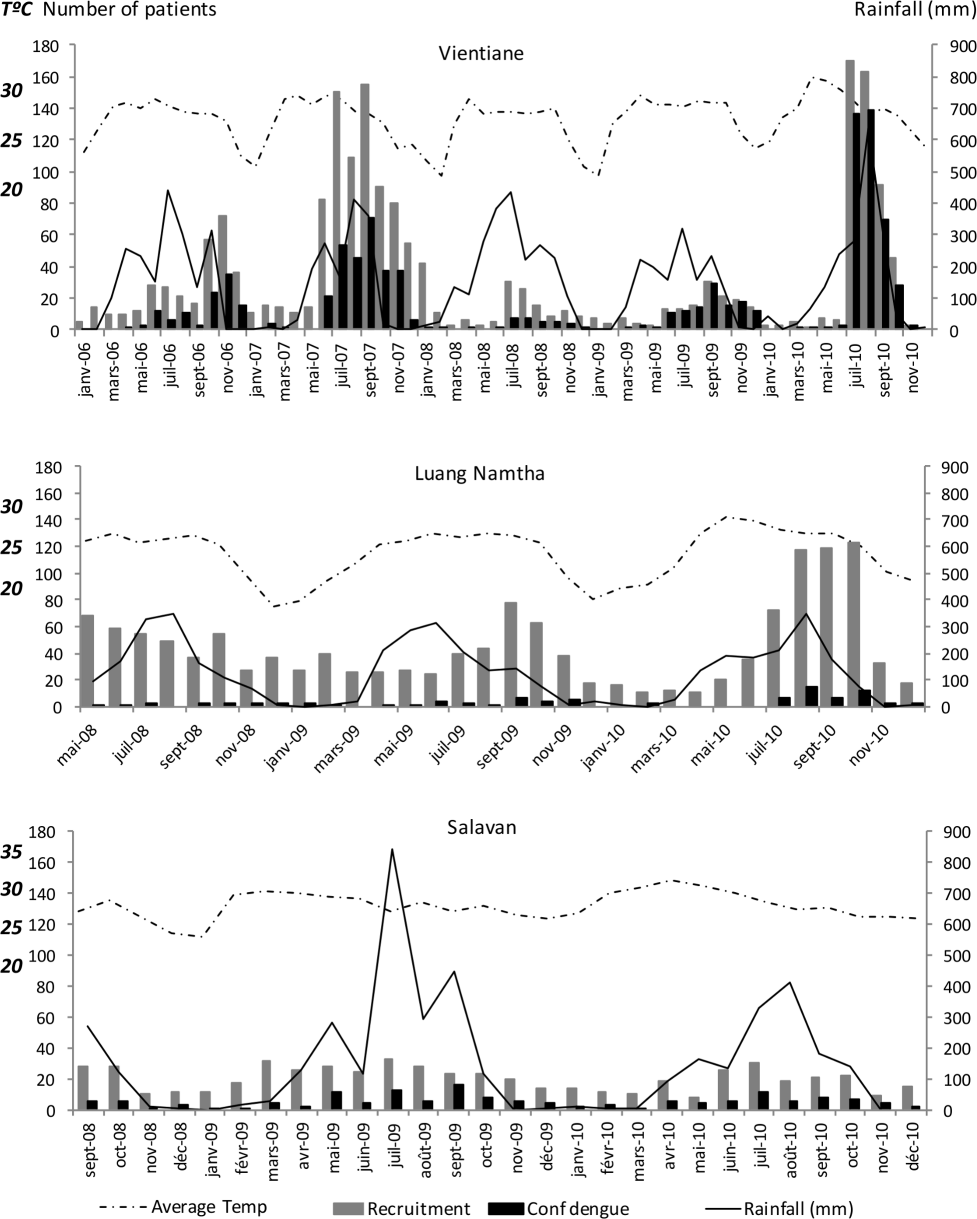
Seasonality of recruited patients and confirmed dengue cases at the three sites. Total: recruited patients. Conf dengue: confirmed dengue cases, Average Temp: monthly mean temperature.

During patient recruitment, monthly mean temperature ranged from 21.4°C to 31.1°C (median 27.5°C) (http://en.tutiempo.net/climate/laos.html) in Vientiane. Clear peaks of rainfall occurred between April and September, corresponding to the rainy season (Fig 2). A higher frequency of recruited and of dengue patients followed rain peaks (Fig 2). Forty-two (42/922, 4.6%) confirmed dengue patients were however detected during the coldest months of the study and outside the rainy season, from December to February.

**Fig 3:**
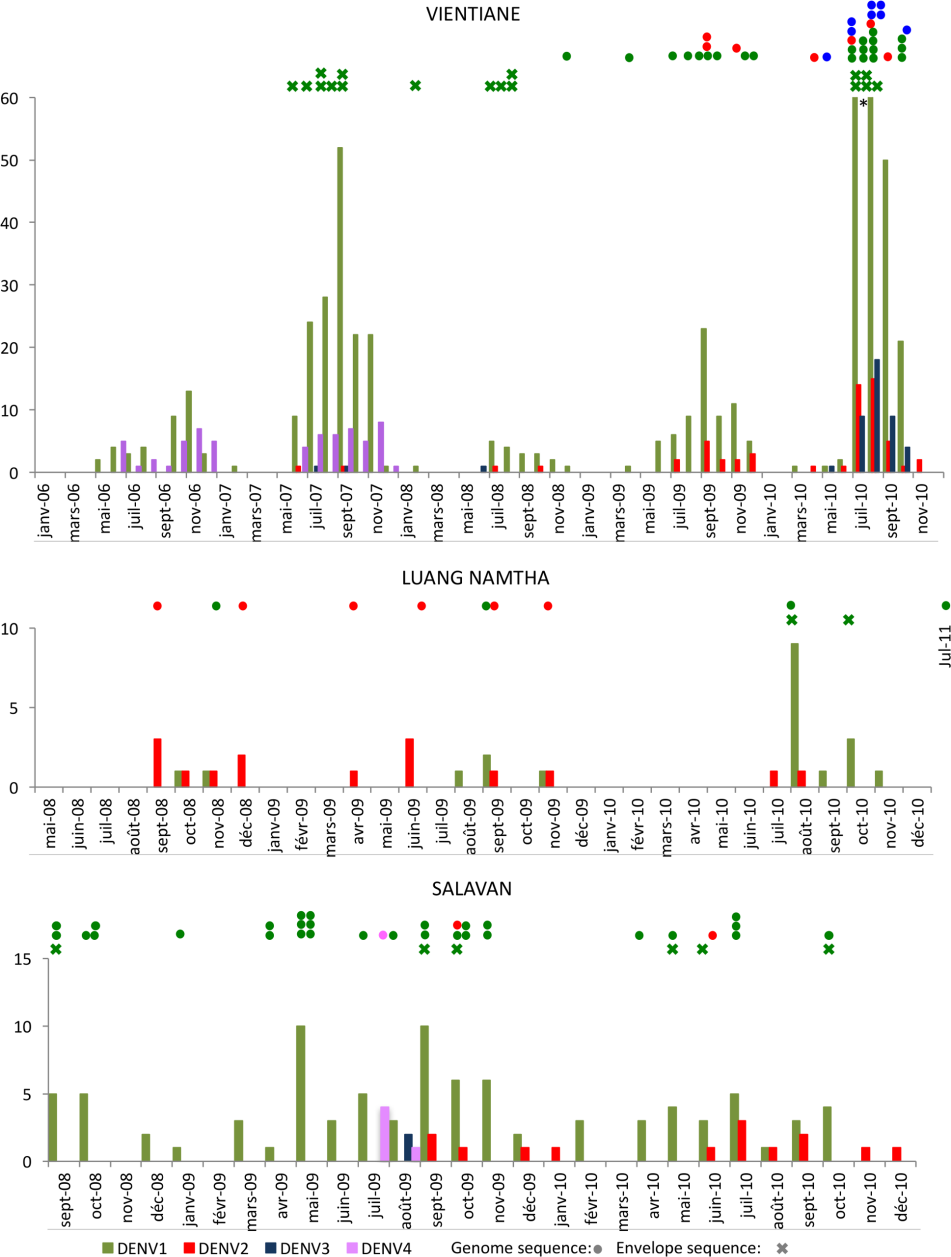
*Dengue virus* serotypes distribution over time at the three sites. Available sequences for each site are indicated with circles for complete CDS and with crosses for envelope on the top of the histograms, including sequences previously published from same locations [23].

A total of 724 samples gave positive results with the DENV real-time RT-PCR (Table 1). All four serotypes were found: DENV-1 in 77.3% (560/724) of RT-PCR positive patients, DENV-2 in 7.6% (55/724), DENV-3 in 6.1% (44/724) and DENV-4 in 8.6% (62/724). DENV-1 was the predominant serotype every year (Fig 3). DENV-4 was only detected in 2006 and 2007. Three patients were found positive for two concurrent serotypes: DENV-1&4 in 2006 and DENV-1&2 in 2008 and 2010. Those patients were not admitted to the hospital with symptoms of severe illness.

### Luang Namtha

In LNT, a total of 1,413 patients were recruited between May 2008 and December 2010 (Table 1). Dengue was confirmed in 90 patients (6.4%) and a further 133 (9.4%) were classified as having a presumptive dengue infection.

During the period of this study, monthly mean temperatures ranged from 18°C to 28.8°C (median 25.8°C), significantly colder than in Vientiane (p<0.001 using Mann Whitney U test) and Salavan (p<0.001 using Mann Whitney U test). As observed in Vientiane, significant decreases in temperature were recorded between December and February with peaks of rainfall between April and September (Fig 2). As in the capital, more patients recruited in the rainy season were diagnosed with dengue although 10/90 (11.1%) confirmed dengue cases were detected during the coldest months of December to February.

A total of 34 samples gave positive results with the DENV real-time RT-PCR (Table 1). DENV-1 and DENV-2 were the only serotypes detected in LNT, in 55.9% (19/34) and 41.2% (14/34), respectively, of all patients. DENV-2 was the predominant serotype in 2008 and 2009 (Fig 3). The year 2010 marked a change and DENV-1 then became significantly more frequent. One patient was found positive for both DENV-1 and DENV-2 in 2008.

#### Salavan

A total of 555 patients were recruited in Salavan between September 2008 and December 2010 (Table 1). Dengue was confirmed in 147 patients (26.4%) while a further 63 (11.3%) were classified as having a presumptive dengue infection.

During the study, monthly mean temperatures ranged from 23.7°C to 31.9°C (median 28.2°C). Rainfall peaks were observed from April to September, coinciding with rainy seasons (Fig 2). More patients had dengue fever during the rainy seasons although no obvious peak was observed. Sixteen (16/147, 10.9%) confirmed dengue cases were detected during the cooler months of December to February.

A total of 109 patients had a positive result by DENV real-time RT-PCR (Table 1). All four serotypes were detected: DENV-1 was found in 80.7% (88/109) of all cases, followed by DENV-2 with 12.8% (14/109) of cases. DENV-1 serotype was the only serotype detected in 2008 (Fig 3) and was dominant in 2009 and 2010. DENV-2 was not detected before September 2009. DENV-3 and DENV-4 were only detected in July 2009 (4 DENV-4 patients) and August 2009 (2 DENV-3 and 1 DENV-4).

### Sequences analysis

The genomes of 75 DENV strains were sequenced: 51 DENV-1 (20 from patients in Vientiane, 2 from LNT (one was from 2011) and 29 from SV), 15 DENV-2 (7 from Vientiane, 6 from LNT and 2 from SV), 8 DENV-3 (all from Vientiane) and 1 DENV-4 (from SV in 2009) (S1 Table, S2 Table and S3 Table). Unfortunately, no samples from 2006 and 2007 were available for sequencing. The dataset includes 43 additional Lao sequences previously published, 33 DENV-1, 3 DENV-2, and 7 DENV-3 [23,36–38] (S4 Table).

#### Recombination

Six recombination events that had never been described before, were detected using RDP and were confirmed by phylogenetic analysis (S5 Table): 1 within serotype 1, 3 within serotype 2 and 2 within serotype 3. No Lao sequence was involved in those recombination events.

#### Phylogenetic analyses

Phylogenetic trees constructed with complete CDS sequences showed that all DENV-1 Lao strains belong to the genotype I, all DENV-2 strains belong to the Asian I genotype, all DENV-3 strains belong to the genotype II and the DENV-4 strain belongs to genotype I (S1 Fig, S2 Fig, S3 Fig and S4 Fig).

Analysis of the envelope trees for each serotype enabled accurate study of strain circulation (S5 Fig, S6 Fig, S7 Fig and S8 Fig). Subtrees with the different clusters that contain Lao strains can be found in S9 Fig, S10 Fig, S11 Fig and S12 Fig.

Dengue virus 1

**Fig 4:**
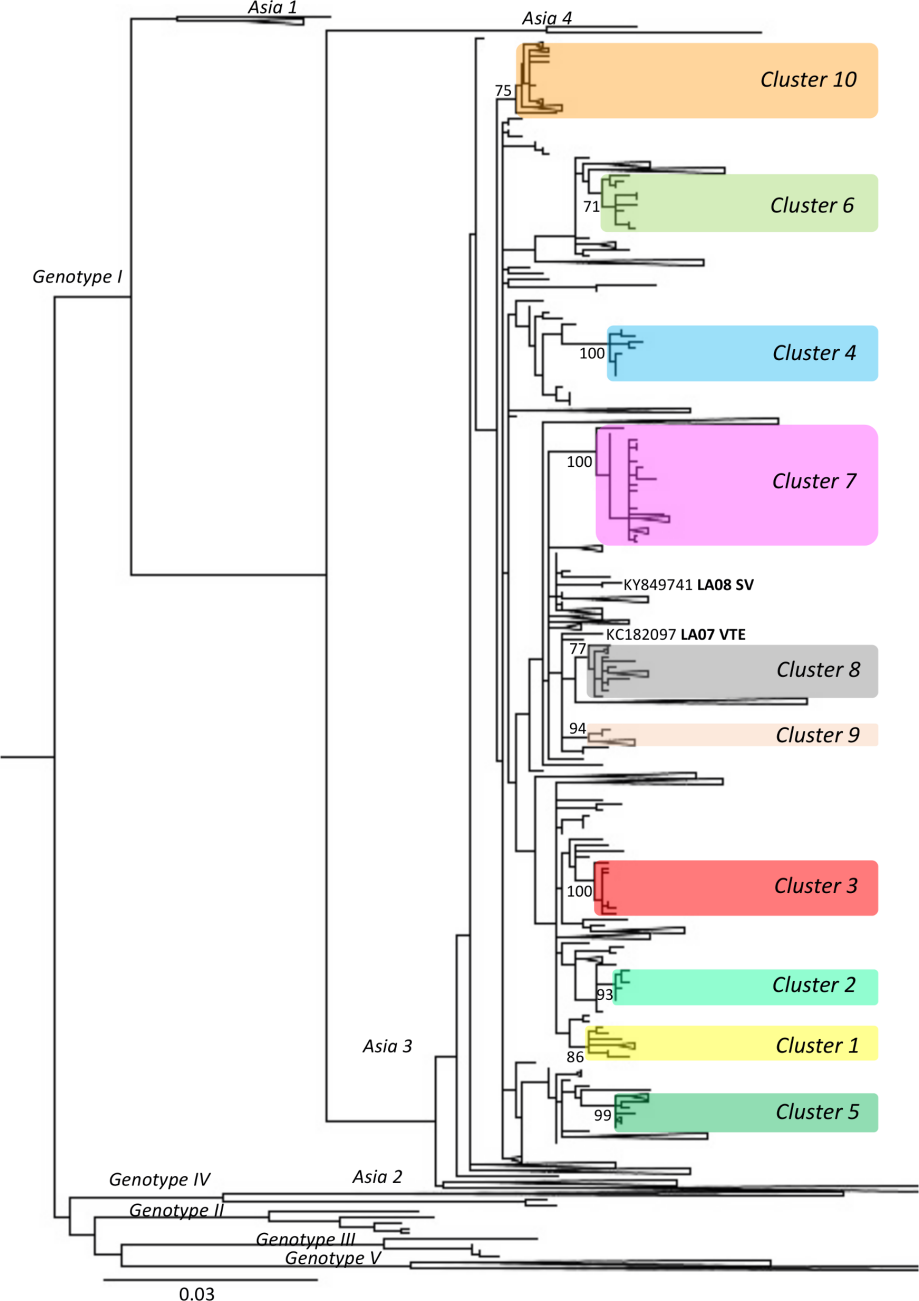
Positions of the 10 clusters containing Lao strains on phylogenetic tree built with DENV-1 envelop sequences. 3,108 DENV-1 envelope sequences, obtained from the European Molecular Biology Laboratory (EMBL) Nucleotide Sequence Database in April 2014, were aligned with the DENV-1 sequences obtained in this study. A tree was built with this dataset using a maximum-likelihood (ML) method (General Time Reversible Model with invariant sites and a gamma distribution of rates across sites) and bootstrap resampling with 500 replicates. 81 Lao strains are dispersed in 10 clusters (bootstrap >70) shaded here in separate colors. The bootstrap values are displayed on the tree only for those clusters. The list of the strains grouped in the different clusters is provided in S6 Table. Two strains from this study do not belong to any of those clusters and are displayed in the tree. The Lao strain from 1996 is not shown on the figure and belongs to Asia 2 clade.

The strain from 1996 belongs to Genotype 1 Asia 2 clade and the 83 other DENV-1 Lao strains are distributed in 10 clusters (sequences that group under a node with a bootstrap >70) within the Asia 3 clade. Two strains could not be included in any clusters: 1 from SV year 2008, closely related to a 2005 Cambodian strain, and 1 from Vientiane year 2007 (Fig 4).

The first 7 clusters were described by Dubot-Pérès *et al*. [23]. Most of the Lao strains are found in cluster 1 and 7, with 28 and 30 strains in each cluster respectively (S9 Fig). The clusters 5, 8, 9 and 10 contain only one Lao strain each (Vientiane 2008, SV 2008, Vientiane 2007, and LNT 2009 respectively). Some clusters contain strains from the same location, cluster 6 from SV (2010), cluster 3 from Vientiane (2007, 2008), cluster 1 from SV (2008, 2009 and 2010 and 1 strain from LNT 2011), cluster 4 from Vientiane and neighboring province (2008, 2009). On the other hand, combinations of strains from SV, LNT and Vientiane are found in clusters 2 and 7 (2008 to 2010). Lao strains from clusters 1, 2, 3, 4 and 10 are closely related to strains from Thailand and those from cluster 7, to a strain that was imported from Thailand to South Korea in 2007. Lao strains from clusters 5 and 6 are related to Cambodian strains, and in cluster 8 and 9 to strains from various Asian countries (Singapore, Sri Lanka, China, Thailand and Taiwan). This suggests Lao strains could originate from these nearby countries.

**Fig 5:**
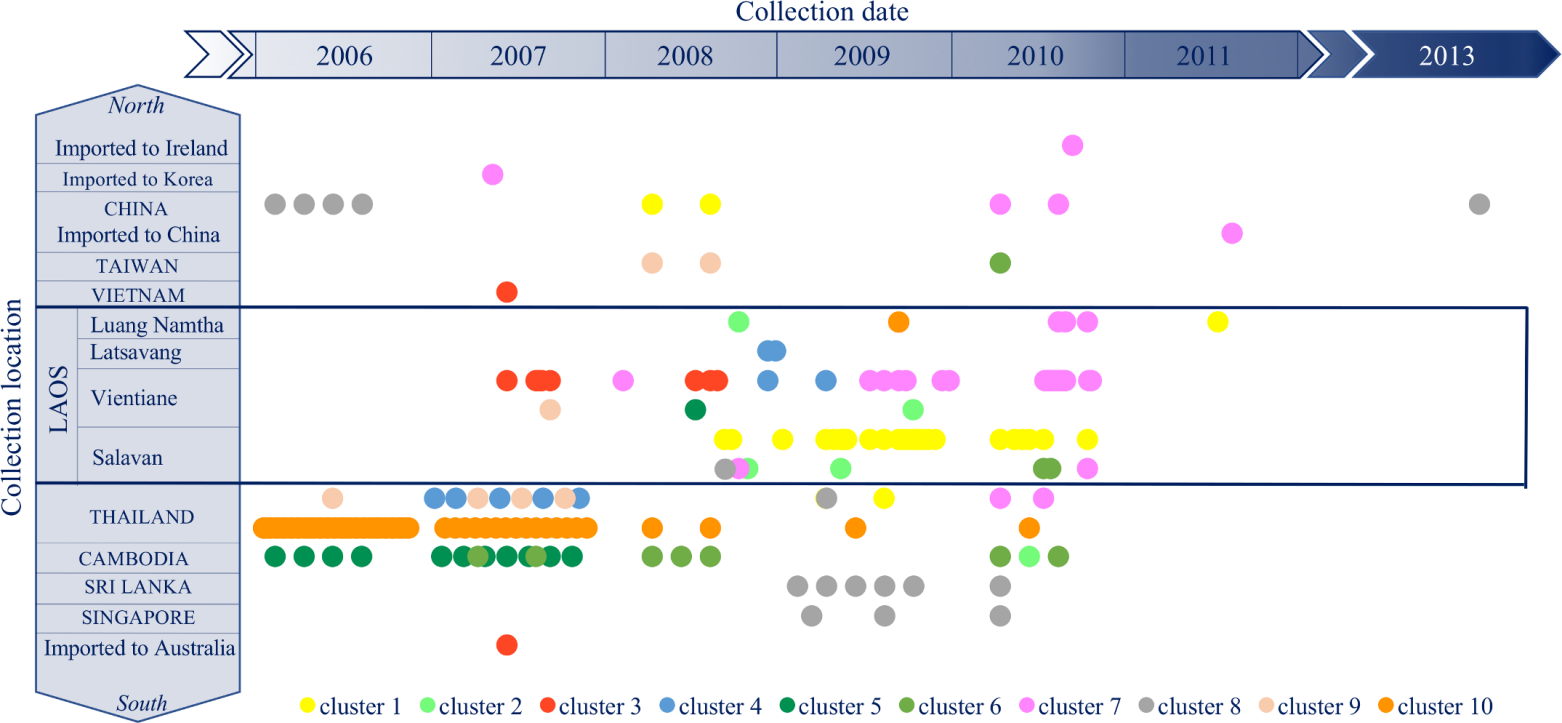
Temporal and geographical distribution of the strains from the ten DENV-1 clusters. 3,108 DENV-1 envelope sequences, obtained from the European Molecular Biology Laboratory (EMBL) Nucleotide Sequence Database in April 2014, were aligned with the DENV-1 Lao sequences obtained in this study. A tree was built (Fig 4) and ten clusters (bootstrap >70) containing the strains from this study were identified. In order to visualize the circulation of all the strains from these different clusters, we plotted each strain according to time of collection and location. Each of the 10 clusters is displayed in a different color: yellow for the strains from cluster # 1, light green for cluster #2, red for cluster #3, blue for cluster #4, dark green for cluster #5, medium green for cluster # 6, pink for cluster #7, grey for cluster #8, light orange for cluster #9 and dark orange for cluster #10. The list of all the strains included in each cluster is provided as S6 Table.

The clusters are displayed in Fig 5 by location of strain isolation over time. Strains from cluster 3 were not detected in Vientiane after mid 2008. Whereas cluster 7 was the main cluster in Vientiane in 2009 and 2010. Strains circulating in Salavan were mainly from cluster 1. Strains from the other clusters were sporadically detected.

Dengue virus 2

Within the Asian I genotype, the 18 DENV-2 Lao strains are grouped in 3 clusters, independently of location and year of isolation (S10 Fig). One strain from LNT 2009 and one strain imported from Laos to Australia (2010) are however outside those clusters. As for DENV-1, the DENV-2 Lao strains are closely related to strains from neighboring countries, mainly from Thailand.

Dengue virus 3

The 8 strains isolated in this study were all from Vientiane 2010 and closely related. They group in a cluster within genotype II (S11 Fig), as described by Lao *et al*. [22], containing strains isolated in Laos and from the Chinese province of Yunnan during the 2012–2013 outbreak [22,38]. The Chinese outbreak has been shown to originate from Laos [38]. The strains published by Lao *et al*. [22] were not included in our dataset since they were submitted to Genbank in November 2014. The 58 genotype II strains are however closely related to our sequences, with more than 99% identity in envelope sequence. Strains from Myanmar from 2005 to 2009 and one strain from Singapore from 2008 are also part of the cluster.

Dengue virus 4

Unfortunately, no sample was available for the 62 Vientiane DENV-4 patients for years 2007 and 2008. Only one strain, from a 2009 SV patient sample, was therefore isolated. This strain groups together with strains from Thailand (2004 to 2010), Cambodia (2001 to 2013) and Vietnam (2011) within genotype I (S12 Fig).

## Discussion

A total of 3,880 patients were recruited in this study and 1,159 (29.9%) were laboratory confirmed as having dengue.

Most of the dengue patients were recruited each year from June to November. This reflects typical infection peaks seen during rainy seasons. Of all confirmed dengue cases, 4.6% in Vientiane and 11% in SV and LNT were however detected between December and February. This is in line with previous findings of active DENV circulation during dry seasons [22,23]. Numbers for Vientiane are however likely to be underestimated since dengue testing was not systematically done during these drier season periods. Interestingly, the number of dengue patients admitted at Mahosot Hospital was significantly higher in 2007 and 2010, in the context of a regional epidemic in 2010 [11,21,39]. The proportion of dengue cases, among recruited patients, was higher in Vientiane (59.8%) than in the other sites (15.8% in Luang Namtha and 37.8% in Salavan). This may be explained by differences in recruitment criteria or physician suspicions, by potentially sub-optimal transport conditions from SV or LNT, but also by environmental criteria since the incidence of DENV is influenced by climate and weather patterns (high temperature and relative humidity have been associated with increased dengue occurrence [40]). The situation in the colder and mountainous Luang Namtha region (northern Laos) is particularly reminiscent of that of the Chinese Yunnan region (adjacent to Laos) where dengue has low frequency [41–43].

DENV-1 and DENV-2 were first reported in 1943 and 1944, respectively, DENV-1 in Japan [44], DENV-2 in Papua New Guinea [45]. DENV-3 and DENV-4 were reported simultaneously in 1956 in the Philippines [46]. DENV-1 has been the most frequently reported serotype in the world since its isolation, followed by DENV-2, DENV-3 and DENV-4. By the end of the 60s, the four DENV serotypes were co-circulating in South-East Asia, where DENV is now hyperendemic in most constituent countries.

In Laos, due to the limited dengue data available, little is known about the dynamics of serotypes circulation. The first serological evidence of the four DENV serotypes circulating in Laos were reported in 1987 [14]. All four serotypes were then detected by RT-PCR among patients in Vientiane in 2004 and 2005 [15]. The National Center for Laboratory and Epidemiology recently reported the results of the Lao National dengue surveillance between 2006 and 2012 [11]. A total of 361 RT-PCR confirmed dengue cases were reported between 2007 and 2012. The four serotypes were detected with DENV-1 as the main serotype until 2011, replaced by DENV-3 in 2012, but it was not stated which provinces the samples came from. Then, in 2013, a large epidemic of DENV-3 was reported in Vientiane [22].

In our study, all four serotypes were detected: DENV-1 from genotype I Asia 3 clade, the predominant clade, DENV-2 from genotype Asian I, DENV-3 from genotype II and DENV-4 from genotype I. Differing dynamics of serotype circulation over time were however observed at the three sites.

DENV-1 was the main serotype detected, in 667 over the 867 DENV RT-PCR positive patients (76.9%). DENV-1 was predominant in Vientiane and SV over the study period, whereas it was rarely detected in LNT before 2010. In contrast, DENV-2 was the main serotype in LNT in 2008 and 2009 whereas it was rarely detected in Vientiane and Salavan before July 2009. Interestingly, similar profile to Vientiane and SV was observed in Thailand with predominant DENV1 and an increase of DENV2 after 2009 [47]. DENV-3 was only detected in five patients before May 2010 (three in Vientiane and two in SV) and was afterwards detected in 41 patients in Vientiane. Large DENV-3 epidemics were then reported in Vientiane in 2012 and 2013 [22]. The eight DENV-3 strains isolated in our study, from Vientiane in 2010, are closely related to the Lao 2012–2013 epidemic strains. This shows there was a local circulation of DENV-3 at least 3 years before the 2013 outbreak. Lao *et al* suggested that this genotype was introduced in Laos in 2011 or before [22]. Our results therefore confirm that the introduction happened prior to 2010. That we did not find any DENV-3 genotype III also supports the suggestion by Lao *et al* [22] that this genotype was only recently introduced into Laos. DENV-4 was not detected in LNT and was limited to five patients in SV although it was the second most common serotype circulating in Vientiane in 2006 (39.7%) and in 2007 (18.5%), then was not detected in the following years in Laos. DENV-4 probably came from Thailand where peaks of cases were reported in 2005 and 2006. DENV-4 infections have rarely been detected in the surrounding countries [48,49], perhaps due to the serotype being less prevalent or the infections being subclinical and less severe.

Active DENV circulation is indeed likely to occur between Laos and neighboring countries, probably more intensively with Thailand. This country has important commercial ties with Laos, facilitated by a long border with multiple ports of entry and similarities in language, which all allow multiple concurrent introductions of new strains. This is supported by our phylogenic analyses showing a distribution of Lao strains from the same periods in different clusters. In addition, DENV circulation throughout Laos may also be important. Indeed, we observed some DENV-1 and DENV-2 strains grouping in clusters independently of locations and years of isolation. The 83 available Lao envelope sequences for DENV-1 (when excluding the 1996 strain from Asia 2 clade) suggest that some strains are maintained over long periods of time after their introduction, whereas others are only sporadically detected. DENV-1 strains were distributed in 10 clusters, with 2 additional strains not fitting in any clusters. Clusters 1 and 7 were predominant since they were maintained in SV and in Vientiane for several years. Cluster 3 seems to have been predominant in Vientiane before the establishment of Cluster 7. Results from remaining clusters suggest frequent introduction events from neighboring countries (Fig 5), with no sustainable maintenance over time or with a “silent” circulation inducing undetected mild or asymptomatic infections. Further investigation of clinical data would prove very useful in understanding differences in possible strain-associated pathogenicity or to confirm the hypothesis that propagation of more virulent strains are more visible to public health surveillance [50]. The study of asymptomatic infections would provide evidence as to whether there are specific populations of strains that are actively circulating “silently”.

Our study provides a picture of the complexity of dengue epidemiology in Laos, with circulation dynamics varying over time according to the serotype and location. Indeed, important variations were observed between the capital of Vientiane, located on the Thai border and connected by flights and road to an increasing number of countries, and the remoter rural areas of Salavan and Luang Namtha. Dengue epidemiology will become more complex with time as tourism and commercial traffic with neighboring countries increase. It therefore is of great importance to develop and strengthen a year-round nation-wide surveillance network, particularly because DENV circulation during inter-epidemic periods plays a crucial part in the onset and the course of the subsequent epidemics. Long term studies are needed in order to determine if periodic multi-annual cycles of dengue epidemic exist in Laos such as observed in Vietnam and Thailand [48,51]. Switches in phylogenetic lineages within one serotype could have important implications as it is often associated with changes in disease severity and incidence. Obtaining more epidemiological as well as virological data will also be crucial for the country for when dengue vaccines becomes available; funding for such a vaccine would need to be justified [52]. This prove to be challenging in developing countries such as Laos, because laboratory facilities capable of confirming dengue cases only exist in the capital city. Innovation to improve simple field collection techniques, such as filter paper or Rapid Diagnosis Tests, to enable surveillance in remote rural Asia is hence needed [53–59].

## Supporting information

S1 Checklist STROBE checklist. (DOC)

S1 Table List of DENV strains sequenced in this study from Vientiane. (DOCX)

S2 Table List of DENV strains sequenced in this study from Luang Namtha. (DOCX)

S3 Table List of DENV strains sequenced in this study from Salavan. (DOCX)

S4 Table Previously published Lao sequences included in the dataset. (DOCX)

S5 Table Evidence for recombination events detected between dengue viruses. Each alignment of complete CDS for the 4 serotypes was submitted to the Recombination Detection Program (RDP) software, version 4 [29]. RDP, GENCONV and MAXCHI methods were used for primary screening and BOOTSCAN and SISCAN methods were used to check for recombination signals. For optimal recombination detection, the automask procedure was selected. Recombination events with an average p-value with RDP lower than E-10 were selected for downstream phylogenetic analyses. The Mega 6.06 software with Kimura-2 model, bootstrap resampling with 500 replicates, was used to build neighbor-joining trees from the alignment used for the RDP analysis. Two trees were produced and compared for each recombination event: one using sequences located between the putative recombination breakpoint positions and a second one which excluded the putative recombinant region. The confirmed recombination events that had not been published before are provided in this table.
(DOCX)

S6 Table List of the DENV-1 strains grouping in the ten Lao clusters. The tree built with the alignment of the 3,108 DENV-1 envelope sequences downloaded from the EMBL database in April 2014 and the sequences from this study allowed the identification of 10 clusters (bootstrap >70) containing Lao strains. Genbank accession numbers of all strains from those 10 clusters are listed in this table.
(DOCX)

S1 Fig Phylogenetic trees built with complete DENV-1 CDS sequences. 1,373 DENV-1 complete CDS downloaded from the EMBL database in April 2014 were aligned with the DENV-1 sequences from this study. The tree was built using the maximum-likelihood (ML) method (General Time Reversible Model with invariant sites and a gamma distribution of rates across sites) and bootstrap resampling with 500 replicates on the RAXML8.0.0 software. Bootstrap values are only displayed for the genotype nodes. Genotypes distribution is illustrated by color-shaded boxes. Red crosses indicate the location of the strains sequenced in this study.
(TIFF)

S2 Fig Phylogenetic trees built with complete DENV-2 CDS sequences. 998 DENV-2 complete CDS downloaded from the EMBL database in April 2014 were aligned with the DENV-2 sequences from this study. The tree was built using a maximum-likelihood (ML) method (General Time-Reversible Model with gamma distributed rates across sites) and bootstrap resampling with 500 replicates on the RAXML8.0.0 software. Bootstrap values are only displayed for the genotype nodes. Genotypes (Sylvatic, American, Cosmopolitan, Asian I and II, Asian/American) distribution is illustrated by color-shaded boxes. Red crosses indicate the location of the strains sequenced in this study.
(TIFF)

S3 Fig Phylogenetic trees built with complete DENV-3 CDS sequences. 670 DENV-3 complete CDS downloaded from the EMBL database in April 2014 were aligned with the DENV-3 sequences from this study. The tree was built using a maximum-likelihood (ML) method (Tamura-Nei model with gamma distributed rates across sites) and bootstrap resampling with 500 replicates on the RAXML8.0.0 software. Bootstrap values are only displayed for the genotype nodes. Genotypes distribution is illustrated color-shaded boxes. Red crosses indicate the location of the strains sequenced in this study.
(TIFF)

S4 Fig Phylogenetic trees built with complete DENV-4 CDS sequences. 123 DENV-4 complete CDS downloaded from the EMBL database in April 2014 were aligned with the DENV-4 sequences from this study. The tree was built using a maximum-likelihood (ML) method (General Time Reversible Model with invariant sites and a gamma distribution of rates across sites) and bootstrap resampling with 500 replicates on the RAXML8.0.0 software. Bootstrap values are only displayed for the genotype nodes. Genotypes distribution is illustrated by color-shaded boxes. Red crosses indicate the location of the strains sequenced in this study.
(TIFF)

S5 Fig Phylogenetic tree built with DENV-1 envelop sequences. 3,108 DENV-1 envelope sequences downloaded from the EMBL database in April 2014 were aligned with the DENV-1 sequences from this study. The tree was built using a maximum-likelihood (ML) method (General Time Reversible Model with invariant sites and a gamma distribution of rates across sites) and bootstrap resampling with 500 replicates on the RAXML8.0.0 software. Bootstrap values are only displayed for the genotype nodes. Genotypes distribution and clades Asia 1 to 4 within Genotype I are illustrated by color-shaded boxes. Six subtrees containing Lao strains were extracted from this tree and displayed in more details in S9 Fig. The orange circle indicates the location of the 1996 Lao strain.
(TIFF)

S6 Fig Phylogenetic tree built with DENV-2 envelop sequences. 3,067 DENV-2 envelop sequences downloaded from the EMBL database in April 2014 were aligned with the DENV-2 sequences from this study. The tree was built using a maximum-likelihood (ML) method (General Time-Reversible Model with gamma distributed rates across sites) and bootstrap resampling with 500 replicates on the RAXML8.0.0 software. Bootstrap values are only displayed for the genotype nodes. Genotypes (Sylvatic, American, Cosmopolitan, Asian I and II, Asian/American) distribution is illustrated by color-shaded boxes. A subtree containing Lao strains was extracted from this tree and displayed in details in S10 Fig.
(TIFF)

S7 Fig Phylogenetic tree built with DENV-3 envelop sequences. 1,817 DENV-3 envelop sequences downloaded from the EMBL database in April 2014 were aligned with the DENV-3 sequences from this study. The tree was built using a maximum-likelihood (ML) method (Tamura-Nei model with gamma distributed rates across sites) and bootstrap resampling with 500 replicates on the RAXML8.0.0 software. Bootstrap values are only displayed for the genotype nodes. Genotypes distribution is illustrated by color-shaded boxes. A subtree containing Lao strains was extracted from this tree and displayed in details in S11 Fig.
(TIFF)

S8 Fig Phylogenetic tree built with DENV-4 envelop sequences. 859 DENV-4 envelop sequences downloaded from the EMBL database in April 2014 were aligned with the DENV-4 sequences from this study. The tree was built using a maximum-likelihood (ML) method (General Time Reversible Model with invariant sites and a gamma distribution of rates across sites) and bootstrap resampling with 500 replicates on the RAXML8.0.0 software. Bootstrap values are only displayed for the genotype nodes. Genotypes distribution is illustrated by color-shaded boxes. A subtree containing Lao strains was extracted from this tree and displayed in details in S12 Fig.
(TIFF)

S9 Fig Subtrees from phylogenic tree built with DENV-1 envelope sequences. Six subtrees containing Lao strains were extracted from the tree built with the complete data set of DENV-1 envelop sequences displayed in S5 Fig. Only bootstrap values over 70 are reported on the figure. The Lao strains are dispersed in ten clusters (bootstrat>70): clusters 1, 2 and 3 in subtree 1 (A); cluster 4 and 5 in subtrees 2 and 3 respectively (B); clusters 6 and 10 in subtrees 4 and 5 respectively (C); clusters 7, 8 and 9 in subtree 6 (D). Strains from Vientiane are indicated by diamonds, strains from LNT by squares and strains from SV by triangles. Strains isolated in 2007 are in yellow, 2008 in orange, 2009 in blue, 2010 in green, and 2011 in pink. Other Lao strains are indicated by brown circles. Lao strains from imported cases are indicated by black circles. Strains are indicated by Genbank accession number followed by country (ISO code) and year of isolation. For the nodes that have been collapsed for simplification purposes (shown by triangles), descendant strains are only described by country and years. The province of origin is indicated for the strains from this study (VTE: Vientiane, SV: Salavan, LNT: Luang Namtha).
(TIF)

S10 Fig Subtree from phylogenic tree built with DENV-2 envelope sequences. A subtree containing Lao strains was extracted from the tree built with the complete data set of DENV-2 envelop sequences displayed in S6 Fig. Only bootstrap values over 70 are reported on the figure. The Lao strains are dispersed in three clusters (bootstrat>70). Strains from Vientiane are indicated by diamonds, strains from LNT by squares and strains from SV by triangles. Strains isolated in 2008 are in orange, 2009 in blue, and 2010 in green. Lao strains from imported cases are shown by black circles. Genbank accession numbers followed by country (ISO code) and year of isolation are provided for each strain. For the nodes that have been collapsed for simplification purposes (shown by triangles), descendant strains are only described by country and years. The province of origin is indicated for the strains from this study (VTE: Vientiane, SV: Salavan, LNT: Luang Namtha).
(TIFF)

S11 Fig Subtree from phylogenic tree built with DENV-3 envelope sequences. A subtree containing Lao strains was extracted from the tree built with the complete data set of DENV-3 envelop sequences displayed in S7 Fig. Only bootstrap values over 70 are reported on the figure. The Lao strains are grouped in one cluster (bootstrat = 99). All the strains from this study were from 2010 and from Vientiane. They are displayed by green diamonds. Other Lao strains are shown by brown circles. Lao strains from imported cases are indicated by black circles. Genbank accession numbers, followed by country (ISO code) and year of isolation are provided for each strain. For the nodes that have been collapsed for simplification purposes (shown by triangles), descendant strains are only described by country and years. The province of origin is indicated for the strains from this study (VTE: Vientiane).
(TIFF)

S12 Fig Subtree from phylogenic tree built with DENV-4 envelope sequences. A subtree containing the Lao strain was extracted from the tree built with the complete data set of DENV-4 envelope sequences displayed in S8 Fig. The Lao strain from this study was from Salavan from 2009 and is shown by a blue triangle. Genbank accession numbers followed by country (ISO code) and year of isolation are provided for each strain. For the nodes that have been collapsed for simplification purposes (shown by triangles), descendant strains are only described by country and years. The province of origin is indicated for the strain from this study (SV: Salavan).
(TIFF)

## References

[1] WHO Dengue Haemorrhagic fever: diagnosis, treatment, prevention and control, 2nd edition. WHO; 1997.

[2] WHO. Dengue: guidelines for diagnosis, treatment, prevention and control—New edition WHO; 2009. 23762963

[3] Dejnirattisai W, Jumnainsong A, Onsirisakul N, Fitton P, Vasanawathana S, Limpitikul W, et al Cross-reacting antibodies enhance dengue virus infection in humans. Science. 2010;328: 745–748. doi: 10.1126/science.1185181 20448183 10.1126/science.1185181 PMC3837288

[4] Fox A, Le NMH, Simmons CP, Wolbers M, Wertheim HFL, Pham TK, et al Immunological and viral determinants of dengue severity in hospitalized adults in Ha Noi, Viet Nam. PLoS Negl Trop Dis. 2011;5: e967 doi: 10.1371/journal.pntd.0000967 21390156 10.1371/journal.pntd.0000967 PMC3046970

[5] Vu TTH, Holmes EC, Duong V, Nguyen TQ, Tran TH, Quail M, et al Emergence of the Asian 1 genotype of dengue virus serotype 2 in viet nam: in vivo fitness advantage and lineage replacement in South-East Asia. PLoS Negl Trop Dis. 2010;4: e757 doi: 10.1371/journal.pntd.0000757 20651932 10.1371/journal.pntd.0000757 PMC2907417

[6] Messina JP, Brady OJ, Scott TW, Zou C, Pigott DM, Duda KA, et al Global spread of dengue virus types: mapping the 70 year history. Trends Microbiol. 2014;22: 138–146. doi: 10.1016/j.tim.2013.12.011 24468533 10.1016/j.tim.2013.12.011 PMC3946041

[7] Bhatt S, Gething PW, Brady OJ, Messina JP, Farlow AW, Moyes CL, et al The global distribution and burden of dengue. Nature. 2013;496: 504–507. doi: 10.1038/nature12060 23563266 10.1038/nature12060 PMC3651993

[8] WHO. Dengue and dengue haemorrhagic fever. Fact sheet N°117. [Internet]. 2015. Available: http://www.who.int/mediacentre/factsheets/fs117/en/

[9] Tang Y, Rodpradit P, Chinnawirotpisan P, Mammen MP, Li T, Lynch JA, et al Comparative analysis of full-length genomic sequences of 10 dengue serotype 1 viruses associated with different genotypes, epidemics, and disease severity isolated in Thailand over 22 years. Am J Trop Med Hyg. 2010;83: 1156–1165. doi: 10.4269/ajtmh.2010.10-0052 21036855 10.4269/ajtmh.2010.10-0052 PMC2963987

[10] 2015 population census [Internet]. Lao Statistic Bureau; Available: http://www.lsb.gov.la/en/index.php

[11] Khampapongpane B, Lewis HC, Ketmayoon P, Phonekeo D, Somoulay V, Khamsing A, et al National dengue surveillance in the Lao People’s Democratic Republic, 2006–2012: epidemiological and laboratory findings. West Pac Surveill Response J WPSAR. 2014;5: 7–13. doi: 10.5365/WPSAR.2014.5.1.001 24734212 10.5365/WPSAR.2014.5.1.001 PMC3984965

[12] Mayxay M, Phetsouvanh R, Moore CE, Chansamouth V, Vongsouvath M, Sisouphone S, et al Predictive diagnostic value of the tourniquet test for the diagnosis of dengue infection in adults. Trop Med Int Health TM IH. 2011;16: 127–133. doi: 10.1111/j.1365-3156.2010.02641.x 20958892 10.1111/j.1365-3156.2010.02641.x PMC3073123

[13] DengueNet [Internet]. WHO; 2016. Available: http://apps.who.int/globalatlas/default.asp

[14] Fukunaga T, Phommasack B, Bounlu K, Saito M, Tadano M, Makino Y, et al Epidemiological situation of dengue infection in Lao P.D.R. Trop Med. 1993;35: 219–227.

[15] Blacksell SD, Bell D, Kelley J, Mammen MP, Gibbons RV, Jarman RG, et al Prospective study to determine accuracy of rapid serological assays for diagnosis of acute dengue virus infection in Laos. Clin Vaccine Immunol CVI. 2007;14: 1458–1464. doi: 10.1128/CVI.00482-06 17715330 10.1128/CVI.00482-06 PMC2168183

[16] Blacksell SD, Mammen MP, Thongpaseuth S, Gibbons RV, Jarman RG, Jenjaroen K, et al Evaluation of the Panbio dengue virus nonstructural 1 antigen detection and immunoglobulin M antibody enzyme-linked immunosorbent assays for the diagnosis of acute dengue infections in Laos. Diagn Microbiol Infect Dis. 2008;60: 43–49. doi: 10.1016/j.diagmicrobio.2007.07.011 17889487 10.1016/j.diagmicrobio.2007.07.011

[17] Hiscox A, Winter CH, Vongphrachanh P, Sisouk T, Somoulay V, Phompida S, et al Serological investigations of flavivirus prevalence in Khammouane Province, Lao People’s Democratic Republic, 2007–2008. Am J Trop Med Hyg. 2010;83: 1166–1169. doi: 10.4269/ajtmh.2010.09-0480 21036856 10.4269/ajtmh.2010.09-0480 PMC2963988

[18] Vallée J, Dubot-Pérès A, Ounaphom P, Sayavong C, Bryant JE, Gonzalez J-P. Spatial distribution and risk factors of dengue and Japanese encephalitis virus infection in urban settings: the case of Vientiane, Lao PDR. Trop Med Int Health TM IH. 2009;14: 1134–1142. doi: 10.1111/j.1365-3156.2009.02319.x 19563430 10.1111/j.1365-3156.2009.02319.x

[19] Mayxay M, Castonguay-Vanier J, Chansamouth V, Dubot-Pérès A, Paris DH, Phetsouvanh R, et al Causes of non-malarial fever in Laos: a prospective study. Lancet Glob Health. 2013;1: e46–54. doi: 10.1016/S2214-109X(13)70008-1 24748368 10.1016/S2214-109X(13)70008-1 PMC3986032

[20] Mayxay M, Sengvilaipaseuth O, Chanthongthip A, Dubot-Pérès A, Rolain J-M, Parola P, et al Causes of Fever in Rural Southern Laos. Am J Trop Med Hyg. 2015;93: 517–520. doi: 10.4269/ajtmh.14-0772 26149859 10.4269/ajtmh.14-0772 PMC4559689

[21] Prasith N, Keosavanh O, Phengxay M, Stone S, Lewis HC, Tsuyuoka R, et al Assessment of gender distribution in dengue surveillance data, the Lao People’s Democratic Republic. West Pac Surveill Response J WPSAR. 2013;4: 17–24. doi: 10.5365/WPSAR.2012.3.4.020 24015367 10.5365/WPSAR.2012.3.4.020 PMC3762968

[22] Lao M, Caro V, Thiberge J-M, Bounmany P, Vongpayloth K, Buchy P, et al Co-circulation of dengue virus type 3 genotypes in Vientiane capital, Lao PDR. PloS One. 2014;9: e115569 doi: 10.1371/journal.pone.0115569 25551768 10.1371/journal.pone.0115569 PMC4281081

[23] Dubot-Pérès A, Vongphrachanh P, Denny J, Phetsouvanh R, Linthavong S, Sengkeopraseuth B, et al An epidemic of dengue-1 in a remote village in rural Laos. PLoS Negl Trop Dis. 2013;7: e2360 doi: 10.1371/journal.pntd.0002360 23951379 10.1371/journal.pntd.0002360 PMC3738459

[24] Ninove L, Nougairede A, Gazin C, Thirion L, Delogu I, Zandotti C, et al RNA and DNA bacteriophages as molecular diagnosis controls in clinical virology: a comprehensive study of more than 45,000 routine PCR tests. PloS One. 2011;6: e16142 doi: 10.1371/journal.pone.0016142 21347398 10.1371/journal.pone.0016142 PMC3036576

[25] Leparc-Goffart I, Baragatti M, Temmam S, Tuiskunen A, Moureau G, Charrel R, et al Development and validation of real-time one-step reverse transcription-PCR for the detection and typing of dengue viruses. J Clin Virol Off Publ Pan Am Soc Clin Virol. 2009;45: 61–66. doi: 10.1016/j.jcv.2009.02.010 19345140 10.1016/j.jcv.2009.02.010

[26] 2012 Case Definitions: Nationally Notifiable Conditions Infectious and Non-Infectious Case Atlanta, GA: Centers for Disease Control and Prevention; 2012.

[27] Baronti C, Piorkowski G, Leparc-Goffart I, de Lamballerie X, Dubot-Pérès A. Rapid next-generation sequencing of dengue, EV-A71 and RSV-A viruses. J Virol Methods. 2015;226: 7–14. doi: 10.1016/j.jviromet.2015.09.004 26376168 10.1016/j.jviromet.2015.09.004

[28] Thompson JD, Gibson TJ, Plewniak F, Jeanmougin F, Higgins DG. The CLUSTAL_X windows interface: flexible strategies for multiple sequence alignment aided by quality analysis tools. Nucleic Acids Res. 1997;25: 4876–4882. 9396791 10.1093/nar/25.24.4876 PMC147148

[29] Martin DP, Murrell B, Golden M, Khoosal A, Muhire B. RDP4: Detection and analysis of recombination patterns in virus genomes. Virus Evol. 2015;1: vev003–vev003. doi: 10.1093/ve/vev003 27774277 10.1093/ve/vev003 PMC5014473

[30] Martin DP, Posada D, Crandall KA, Williamson C. A modified bootscan algorithm for automated identification of recombinant sequences and recombination breakpoints. AIDS Res Hum Retroviruses. 2005;21: 98–102. doi: 10.1089/aid.2005.21.98 15665649 10.1089/aid.2005.21.98

[31] Padidam M, Sawyer S, Fauquet CM. Possible emergence of new geminiviruses by frequent recombination. Virology. 1999;265: 218–225. doi: 10.1006/viro.1999.0056 10600594 10.1006/viro.1999.0056

[32] Maynard Smith J. Analysing the mosaic structure of genes. J Mol Evol. 1992;34: 126–129. 1556748 10.1007/BF00182389

[33] Gibbs MJ, Armstrong JS, Gibbs AJ. Sister-scanning: a Monte Carlo procedure for assessing signals in recombinant sequences. Bioinforma Oxf Engl. 2000;16: 573–582. 10.1093/bioinformatics/16.7.573 11038328

[34] Tamura K, Stecher G, Peterson D, Filipski A, Kumar S. MEGA6: Molecular Evolutionary Genetics Analysis version 6.0. Mol Biol Evol. 2013;30: 2725–2729. doi: 10.1093/molbev/mst197 24132122 10.1093/molbev/mst197 PMC3840312

[35] Stamatakis A. RAxML version 8: a tool for phylogenetic analysis and post-analysis of large phylogenies. Bioinforma Oxf Engl. 2014;30: 1312–1313. doi: 10.1093/bioinformatics/btu033 24451623 10.1093/bioinformatics/btu033 PMC3998144

[36] Huang J-H, Su C-L, Yang C-F, Liao T-L, Hsu T-C, Chang S-F, et al Molecular characterization and phylogenetic analysis of dengue viruses imported into Taiwan during 2008–2010. Am J Trop Med Hyg. 2012;87: 349–358. doi: 10.4269/ajtmh.2012.11-0666 22855770 10.4269/ajtmh.2012.11-0666 PMC3414576

[37] Warrilow D, Northill JA, Pyke AT. Sources of dengue viruses imported into Queensland, australia, 2002–2010. Emerg Infect Dis. 2012;18: 1850–1857. doi: 10.3201/eid1811.120014 23092682 10.3201/eid1811.120014 PMC3559152

[38] Guo X, Yang H, Wu C, Jiang J, Fan J, Li H, et al Molecular Characterization and Viral Origin of the First Dengue Outbreak in Xishuangbanna, Yunnan Province, China, 2013. Am J Trop Med Hyg. 2015;93: 390–393. doi: 10.4269/ajtmh.14-0044 26078324 10.4269/ajtmh.14-0044 PMC4530767

[39] Undurraga EA, Halasa YA, Shepard DS. Use of expansion factors to estimate the burden of dengue in Southeast Asia: a systematic analysis. PLoS Negl Trop Dis. 2013;7: e2056 doi: 10.1371/journal.pntd.0002056 23437407 10.1371/journal.pntd.0002056 PMC3578761

[40] Pham HV, Doan HTM, Phan TTT, Minh NNT. Ecological factors associated with dengue fever in a Central Highlands province, Vietnam. BMC Infect Dis. 2011;11: 172 doi: 10.1186/1471-2334-11-172 21679398 10.1186/1471-2334-11-172 PMC3126728

[41] Gao X, Nasci R, Liang G. The neglected arboviral infections in mainland China. PLoS Negl Trop Dis. 2010;4: e624 doi: 10.1371/journal.pntd.0000624 20436960 10.1371/journal.pntd.0000624 PMC2860493

[42] Liu W, Zuo L, Zhou Y. The distribution of DEN infected people in Dushan and Xingyi area of Yunnan-Guizhou Plateau, China. Cell Mol Immunol. 2006;3: 473–476. 17257502

[43] Wu W, Bai Z, Zhou H, Tu Z, Fang M, Tang B, et al Molecular epidemiology of dengue viruses in southern China from 1978 to 2006. Virol J. 2011;8: 322 doi: 10.1186/1743-422X-8-322 21703015 10.1186/1743-422X-8-322 PMC3138434

[44] Hotta S. Experimental studies on dengue. I. Isolation, identification and modification of the virus. J Infect Dis. 1952;90: 1–9. 14888958 10.1093/infdis/90.1.1

[45] Sabin AB. Research on dengue during World War II. Am J Trop Med Hyg. 1952;1: 30–50. 14903434 10.4269/ajtmh.1952.1.30

[46] Hammon WM, Rudnick A, Sather GE. Viruses associated with epidemic hemorrhagic fevers of the Philippines and Thailand. Science. 1960;131: 1102–1103. 14399343 10.1126/science.131.3407.1102

[47] Limkittikul K, Brett J, L’Azou M. Epidemiological trends of dengue disease in Thailand (2000–2011): a systematic literature review. PLoS Negl Trop Dis. 2014;8: e3241 doi: 10.1371/journal.pntd.0003241 25375766 10.1371/journal.pntd.0003241 PMC4222696

[48] Adams B, Holmes EC, Zhang C, Mammen MP, Nimmannitya S, Kalayanarooj S, et al Cross-protective immunity can account for the alternating epidemic pattern of dengue virus serotypes circulating in Bangkok. Proc Natl Acad Sci U S A. 2006;103: 14234–14239. doi: 10.1073/pnas.0602768103 16966609 10.1073/pnas.0602768103 PMC1599940

[49] Recker M, Blyuss KB, Simmons CP, Hien TT, Wills B, Farrar J, et al Immunological serotype interactions and their effect on the epidemiological pattern of dengue. Proc Biol Sci. 2009;276: 2541–2548. doi: 10.1098/rspb.2009.0331 19369266 10.1098/rspb.2009.0331 PMC2684681

[50] Rico-Hesse R. Microevolution and virulence of dengue viruses. Adv Virus Res. 2003;59: 315–341. 14696333 10.1016/s0065-3527(03)59009-1 PMC3045824

[51] Thai KTD, Cazelles B, Nguyen NV, Vo LT, Boni MF, Farrar J, et al Dengue Dynamics in Binh Thuan Province, Southern Vietnam: Periodicity, Synchronicity and Climate Variability. Gubler DJ, editor. PLoS Negl Trop Dis. 2010;4: e747 doi: 10.1371/journal.pntd.0000747 20644621 10.1371/journal.pntd.0000747 PMC2903474

[52] Zorlu G, Fleck F. Dengue vaccine roll-out: getting ahead of the game. Bull World Health Organ. 2011;89: 476–477. doi: 10.2471/BLT.11.030711 21734760 10.2471/BLT.11.030711 PMC3127272

[53] Elliott I, Dittrich S, Paris D, Sengduanphachanh A, Phoumin P, Newton PN. The use of dried cerebrospinal fluid filter paper spots as a substrate for PCR diagnosis of the aetiology of bacterial meningitis in the Lao PDR. Clin Microbiol Infect Off Publ Eur Soc Clin Microbiol Infect Dis. 2013;19: E466–472. doi: 10.1111/1469-0691.12260 23738720 10.1111/1469-0691.12260 PMC4285853

[54] Phommasone K, Sengvilaipaseuth O, de Lamballerie X, Vongsouvath M, Phonemixay O, Blacksell SD, et al Temperature and the field stability of a dengue rapid diagnostic test in the tropics. Am J Trop Med Hyg. 2015;93: 33–39. doi: 10.4269/ajtmh.15-0142 25962773 10.4269/ajtmh.15-0142 PMC4497900

[55] Tricou V, Vu HTT, Quynh NVN, Nguyen CVV, Tran HT, Farrar J, et al Comparison of two dengue NS1 rapid tests for sensitivity, specificity and relationship to viraemia and antibody responses. BMC Infect Dis. 2010;10: 142 doi: 10.1186/1471-2334-10-142 20509940 10.1186/1471-2334-10-142 PMC2895602

[56] Osorio L, Ramirez M, Bonelo A, Villar LA, Parra B. Comparison of the diagnostic accuracy of commercial NS1-based diagnostic tests for early dengue infection. Virol J. 2010;7: 361 doi: 10.1186/1743-422X-7-361 21134275 10.1186/1743-422X-7-361 PMC3016282

[57] Wang SM, Sekaran SD. Early diagnosis of Dengue infection using a commercial Dengue Duo rapid test kit for the detection of NS1, IGM, and IGG. Am J Trop Med Hyg. 2010;83: 690–695. doi: 10.4269/ajtmh.2010.10-0117 20810840 10.4269/ajtmh.2010.10-0117 PMC2929071

[58] Vongsouvath M, Phommasone K, Sengvilaipaseuth O, Kosoltanapiwat N, Chantratita N, Blacksell SD, et al Using Rapid Diagnostic Tests as a Source of Viral RNA for Dengue Serotyping by RT-PCR—A Novel Epidemiological Tool. PLoS Negl Trop Dis. 2016;10: e0004704 doi: 10.1371/journal.pntd.0004704 27159058 10.1371/journal.pntd.0004704 PMC4861341

[59] Bharucha T, Chanthongthip A, Phuangpanom S, Phonemixay O, Sengvilaipaseuth O, Vongsouvath M, et al Pre-cut Filter Paper for Detecting Anti-Japanese Encephalitis Virus IgM from Dried Cerebrospinal Fluid Spots. PLoS Negl Trop Dis. 2016;10: e0004516 doi: 10.1371/journal.pntd.0004516 26986061 10.1371/journal.pntd.0004516 PMC4795698

