# Supplementary material for: Molecular epidemiology of dengue viruses in three provinces of Lao PDR, 2006-2010
Source: PLoS Negl Trop Dis. 2018 Jan 29;12(1):e0006203. doi: 10.1371/journal.pntd.0006203 (PMC5805359; doi:10.1371/journal.pntd.0006203)
Supplement: S1 Table — (DOCX) [file pntd.0006203.s002.docx]

**S1 Table. List of DENV strains sequenced in this study from Vientiane.**

| **Serotype** | **strain** | **location** | **sample date** | **sample type** | **size** | **Genbank accession number** |
| --- | --- | --- | --- | --- | --- | --- |
| DENV-1 | UI14029 | VTE | 9-Apr-09 | culture | 10,675 | KY849745 |
| DENV-1 | UI14612 | VTE | 7-Jul-09 | culture | 10,675 | KY849724 |
| DENV-1 | UI14881 | VTE | 12-Aug-09 | culture | 10,675 | KY849746 |
| DENV-1 | UI15040 | VTE | 3-Sep-09 | culture | 10,675 | KY849725 |
| DENV-1 | UI15219 | VTE | 24-Sep-09 | culture | 10,675 | KY849726 |
| DENV-1 | UI15488 | VTE | 28-Oct-09 | culture | 10,675 | KY849727 |
| DENV-1 | UI15711 | VTE | 4-Dec-09 | culture | 10,675 | KY849728 |
| DENV-1 | UI15814 | VTE | 24-Dec-09 | culture | 10,675 | KY849729 |
| DENV-1 | UI17268 | VTE | 6-Jul-10 | culture | 10,675 | KY849747 |
| DENV-1 | UI17443 | VTE | 18-Jul-10 | culture | 10,675 | KY849730 |
| DENV-1 | UI17517 | VTE | 23-Jul-10 | culture | 10,675 | KY849731 |
| DENV-1 | UI17524 | VTE | 24-Jul-10 | culture | 10,675 | KY849702 |
| DENV-1 | UI17578 | VTE | 27-Jul-10 | culture | 10,675 | KY849732 |
| DENV-1 | UI17968 | VTE | 23-Aug-10 | culture | 10,675 | KY849733 |
| DENV-1 | UI18014 | VTE | 26-Aug-10 | culture | 10,675 | KY849734 |
| DENV-1 | UI18022 | VTE | 26-Aug-10 | culture | 10,675 | KY849735 |
| DENV-1 | UI18058 | VTE | 30-Aug-10 | culture | 10,675 | KY849736 |
| DENV-1 | UI18560 | VTE | 13-Oct-10 | culture | 10,675 | KY849737 |
| DENV-1 | UI18600 | VTE | 18-Oct-10 | culture | 10,675 | KY849738 |
| DENV-1 | UI18608 | VTE | 19-Oct-10 | culture | 10,675 | KY849739 |
| DENV-2 | UI15033 | VTE | 2-Sep-09 | culture | 10,675 | KY849756 |
| DENV-2 | UI15067 | VTE | 7-Sep-09 | serum | 10,674 | KY849764 |
| DENV-2 | UI15560 | VTE | 6-Nov-09 | serum | 10,675 | KY849765 |
| DENV-2 | UI16615 | VTE | 29-Apr-10 | serum | 10,675 | KY849766 |
| DENV-2 | UI17564 | VTE | 26-Jul-10 | serum | 10,675 | KY849767 |
| DENV-2 | UI17827 | VTE | 13-Aug-10 | serum | 10,675 | KY849768 |
| DENV-2 | UI18384 | VTE | 30-Sep-10 | serum | 10,669 | KY849763 |
| DENV-3 | UI16836 | VTE | 26-May-10 | serum | 10,659 | KY849771 |
| DENV-3 | UI17402 | VTE | 25-Jul-10 | serum | 10,659 | KY849773 |
| DENV-3 | UI17583 | VTE | 27-Jul-10 | culture | 10,660 | KY849761 |
| DENV-3 | UI17706 | VTE | 5-Aug-10 | serum | 10,659 | KY849775 |
| DENV-3 | UI17760 | VTE | 9-Aug-10 | serum | 10,645 | KY849769 |
| DENV-3 | UI17816 | VTE | 12-Aug-10 | serum | 10,659 | KY849770 |
| DENV-3 | UI17982 | VTE | 24-Aug-10 | serum | 10,659 | KY849772 |
| DENV-3 | UI18630 | VTE | 21-Oct-10 | serum | 10,660 | KY849774 |
