# Supplementary material for: Molecular epidemiology of dengue viruses in three provinces of Lao PDR, 2006-2010
Source: PLoS Negl Trop Dis. 2018 Jan 29;12(1):e0006203. doi: 10.1371/journal.pntd.0006203 (PMC5805359; doi:10.1371/journal.pntd.0006203)
Supplement: S2 Table — (DOCX) [file pntd.0006203.s003.docx]

**S2 Table. List of DENV strains sequenced in this study from Luang Namtha.**

| **Serotype** | **strain** | **location** | **sample date** | **sample type** | **size** | **Genbank accession number** |
| --- | --- | --- | --- | --- | --- | --- |
| DENV-1 | LNT1660 | LNT | 17-Aug-10 | culture | 10,675 | KY849703 |
| DENV-1 | LNT2313 | LNT | 2-Jul-11 | culture | 10,675 | KY849704 |
| DENV-2 | LNT555 | LNT | 7-Sep-08 | culture | 10,675 | KY849752 |
| DENV-2 | LNT713 | LNT | 6-Dec-08 | culture | 10,676 | KY849759 |
| DENV-2 | LNT866 | LNT | 6-Apr-09 | culture | 10,675 | KY849753 |
| DENV-2 | LNT959 | LNT | 23-Jun-09 | culture | 10,675 | KY849754 |
| DENV-2 | LNT1155 | LNT | 25-Sep-09 | culture | 10,675 | KY849757 |
| DENV-2 | LNT1266 | LNT | 9-Nov-09 | culture | 10,676 | KY849760 |
