# Supplementary material for: Molecular epidemiology of dengue viruses in three provinces of Lao PDR, 2006-2010
Source: PLoS Negl Trop Dis. 2018 Jan 29;12(1):e0006203. doi: 10.1371/journal.pntd.0006203 (PMC5805359; doi:10.1371/journal.pntd.0006203)
Supplement: S3 Table — (DOCX) [file pntd.0006203.s004.docx]

**S3 Table. List of DENV strains sequenced in this study from Salavan.**

| **Serotype** | **strain** | **location** | **sample date** | **sample type** | **size** | **Genbank accession number** |
| --- | --- | --- | --- | --- | --- | --- |
| DENV-1 | SV36 | SV | 10-Sep-08 | culture | 10,675 | KY849740 |
| DENV-1 | SV32 | SV | 11-Sep-08 | culture | 10,675 | KY849705 |
| DENV-1 | SV68 | SV | 6-Oct-08 | culture | 10,675 | KY849741 |
| DENV-1 | SV79 | SV | 15-Oct-08 | culture | 10,676 | KY849749 |
| DENV-1 | SV86 | SV | 22-Oct-08 | culture | 10,675 | KY849706 |
| DENV-1 | SV130 | SV | 27-Jan-09 | culture | 10,675 | KY849707 |
| DENV-1 | SV204 | SV | 10-Apr-09 | culture | 10,675 | KY849708 |
| DENV-1 | SV218 | SV | 30-Apr-09 | culture | 10,675 | KY849709 |
| DENV-1 | SV220 | SV | 4-May-09 | culture | 10,675 | KY849710 |
| DENV-1 | SV224 | SV | 6-May-09 | culture | 10,676 | KY849750 |
| DENV-1 | SV228 | SV | 9-May-09 | culture | 10,675 | KY849711 |
| DENV-1 | SV231 | SV | 12-May-09 | culture | 10,675 | KY849712 |
| DENV-1 | SV232 | SV | 9-May-09 | culture | 10,675 | KY849713 |
| DENV-1 | SV243 | SV | 18-May-09 | culture | 10,675 | KY849714 |
| DENV-1 | SV300 | SV | 18-Jul-09 | culture | 10,675 | KY849742 |
| DENV-1 | SV337 | SV | 11-Aug-09 | culture | 10,673 | KY849701 |
| DENV-1 | SV381 | SV | 26-Sep-09 | culture | 10,675 | KY849715 |
| DENV-1 | SV385 | SV | 28-Sep-09 | culture | 10,675 | KY849716 |
| DENV-1 | SV393 | SV | 13-Oct-09 | culture | 10,675 | KY849748 |
| DENV-1 | SV409 | SV | 23-Oct-09 | culture | 10,675 | KY849717 |
| DENV-1 | SV410 | SV | 22-Oct-09 | culture | 10,675 | KY849718 |
| DENV-1 | SV427 | SV | 4-Nov-09 | culture | 10,675 | KY849719 |
| DENV-1 | SV437 | SV | 12-Nov-09 | culture | 10,675 | KY849720 |
| DENV-1 | SV532 | SV | 17-Apr-10 | culture | 10,675 | KY849721 |
| DENV-1 | SV541 | SV | 5-May-10 | culture | 10,675 | KY849722 |
| DENV-1 | SV582 | SV | 6-Jul-10 | culture | 10,675 | KY849723 |
| DENV-1 | SV594 | SV | 15-Jul-10 | culture | 10,675 | KY849743 |
| DENV-1 | SV602 | SV | 25-Jul-10 | culture | 10,675 | KY849744 |
| DENV-1 | SV671 | SV | 6-Oct-10 | culture | 10,675 | KY849751 |
| DENV-2 | SV397 | SV | 14-Oct-09 | culture | 10,675 | KY849758 |
| DENV-2 | SV576 | SV | 29-Jun-10 | culture | 10,675 | KY849755 |
| DENV-4 | SV296 | SV | 13-Jul-09 | culture | 10,603 | KY849762 |
