# Supplementary material for: Molecular epidemiology of dengue viruses in three provinces of Lao PDR, 2006-2010
Source: PLoS Negl Trop Dis. 2018 Jan 29;12(1):e0006203. doi: 10.1371/journal.pntd.0006203 (PMC5805359; doi:10.1371/journal.pntd.0006203)
Supplement: S4 Table — (DOCX) [file pntd.0006203.s005.docx]

**S4 Table. Previously published Lao sequences included in the dataset.**

| Serotype | **Gnebank** | **Isolation location** | **Isolation date** | **Publication** |
| --- | --- | --- | --- | --- |
| DENV1 | KC172829 | Latsavang | 15-Dec-08 | Dubot-Peres et al 2013 |
| DENV1 | KC172830 | Latsavang | 15-Dec-08 | Dubot-Peres et al 2013 |
| DENV1 | KC172831 | Luang Namtha | 10-Oct-08 | Dubot-Peres et al 2013 |
| DENV1 | KC172832 | Luang Namtha | 16-Sep-09 | Dubot-Peres et al 2013 |
| DENV1 | KC182082 | Luang Namtha | 09-Aug-10 | Dubot-Peres et al 2013 |
| DENV1 | KC182084 | Luang Namtha | 26-Oct-10 | Dubot-Peres et al 2013 |
| DENV1 | KC182085 | Salavan | 09-Sep-08 | Dubot-Peres et al 2013 |
| DENV1 | KC182088 | Salavan | 24-Sep-09 | Dubot-Peres et al 2013 |
| DENV1 | KC182089 | Salavan | 26-Oct-09 | Dubot-Peres et al 2013 |
| DENV1 | KC182091 | Salavan | 15-May-10 | Dubot-Peres et al 2013 |
| DENV1 | KC182092 | Salavan | 14-Jun-10 | Dubot-Peres et al 2013 |
| DENV1 | KC182095 | Salavan | 27-Oct-10 | Dubot-Peres et al 2013 |
| DENV1 | KC182096 | Vientiane | 14-Jun-07 | Dubot-Peres et al 2013 |
| DENV1 | KC182097 | Vientiane | 13-Jul-07 | Dubot-Peres et al 2013 |
| DENV1 | KC182098 | Vientiane | 01-Aug-07 | Dubot-Peres et al 2013 |
| DENV1 | KC182099 | Vientiane | 10-Aug-07 | Dubot-Peres et al 2013 |
| DENV1 | KC182100 | Vientiane | 23-Aug-07 | Dubot-Peres et al 2013 |
| DENV1 | KC182101 | Vientiane | 01-Sep-07 | Dubot-Peres et al 2013 |
| DENV1 | KC182102 | Vientiane | 07-Sep-07 | Dubot-Peres et al 2013 |
| DENV1 | KC182103 | Vientiane | 06-Feb-08 | Dubot-Peres et al 2013 |
| DENV1 | KC182104 | Vientiane | 08-Jul-08 | Dubot-Peres et al 2013 |
| DENV1 | KC182105 | Vientiane | 22-Jul-08 | Dubot-Peres et al 2013 |
| DENV1 | KC182106 | Vientiane | 11-Aug-08 | Dubot-Peres et al 2013 |
| DENV1 | KC182107 | Vientiane | 18-Aug-08 | Dubot-Peres et al 2013 |
| DENV1 | KC172835 | Vientiane | 24-Dec-08 | Dubot-Peres et al 2013 |
| DENV1 | KC182108 | Vientiane | 05-Jul-10 | Dubot-Peres et al 2013 |
| DENV1 | KC182109 | Vientiane | 13-Jul-10 | Dubot-Peres et al 2013 |
| DENV1 | KC182110 | Vientiane | 20-Jul-10 | Dubot-Peres et al 2013 |
| DENV1 | KC182111 | Vientiane | 28-Jul-10 | Dubot-Peres et al 2013 |
| DENV1 | KC182112 | Vientiane | 18-Aug-10 | Dubot-Peres et al 2013 |
| DENV1 | AB003090 |  | 1996 |  |
| DENV1 | JN415509 | Australia | 29-Jun-05 | Warrilow *et al*. 2012 |
| DENV1 | KF926700 | China | 03-Jul-05 |  |
| DENV2 | JN568244 | Australia | 2010 | Warrilow *et al*. 2012 |
| DENV2 | JF9680020 | Taiwan | 2010 | Huang *et al*. 2012 |
| DENV2 | JF9680021 | Taiwan | 2010 | Huang *et al*. 2012 |
| DENV3 | KF816158 | Luangprabang | 2013 | Guo *et al*. 2015 |
| DENV3 | KF816159 | Luangprabang | 2013 | Guo *et al*. 2015 |
| DENV3 | KF816147 | China | 2013 | Guo *et al*. 2015 |
| DENV3 | KF816148 | China | 2013 | Guo *et al*. 2015 |
| DENV3 | KF816162 | China | 2013 | Guo *et al*. 2015 |
| DENV3 | KF816160 | Houayxay | 2013 | Guo *et al*. 2015 |
| DENV3 | KF816161 | Houayxay | 2013 | Guo *et al*. 2015 |
