# Supplementary material for: Molecular epidemiology of dengue viruses in three provinces of Lao PDR, 2006-2010
Source: PLoS Negl Trop Dis. 2018 Jan 29;12(1):e0006203. doi: 10.1371/journal.pntd.0006203 (PMC5805359; doi:10.1371/journal.pntd.0006203)
Supplement: S5 Table — Each alignment of complete CDS for the 4 serotypes was submitted to the Recombination Detection Program (RDP) software, version 4 [29]. RDP, GENCONV and MAXCHI methods were used for primary screening and BOOTSCAN and SISCAN methods were used to check for recombination signals. For optimal recombination detection, the automask procedure was selected. Recombination events with an average p-value with RDP lower than E-10 were selected for downstream phylogenetic analyses. The Mega 6.06 software with Kimura-2 model, bootstrap resampling with 500 replicates, was used to build neighbor-joining trees from the alignment used for the RDP analysis. Two trees were produced and compared for each recombination event: one using sequences located between the putative recombination breakpoint positions and a second one which excluded the putative recombinant region. The confirmed recombination events that had not been published before are provided in this table. (DOCX) [file pntd.0006203.s006.docx]

**S5 Table. Evidence for recombination events detected between dengue viruses.**

| **Sero** | **Recomb** | **Major parent** | **Minor parent** | **Starting breakp** | **Ending breakp** | **Genes involved** | **P value** |
| --- | --- | --- | --- | --- | --- | --- | --- |
| 1 | JF459993 | AY726553 | FJ196841 | 13 * | 1012 | C:E | 1E-58 |
| 3 | DQ675519 | AY858038 | AF317645 | 2453 | 3167 | NS1 | 3,4E-14 |
| 3 | JF295012 | GQ868628 | AY099336 | 5299 | 5671 | NS3 | 1,5E-34 |
| 2 | GQ398282 | GQ398283 | GQ398286 | 6277 | 8453 | NS4A:NS5 | 3,4E-23 |
| 2 | GQ398269 | GQ398282 | AF038402 | 4179 | 6261 | NS2A:NS4A | 4E-69 |
| 2 | AF038402 | AF038403 | DQ181804 | 1 * | 2184 * | C:E | 1,5E-29 |

* No beginning breakpoint identified, hypothetical breakpoint determined according to its position in the alignment (given by RDP)

Sero: serotype all strains involved in the recombination event are belonging to. Recomb: recombinant srain. Starting breakp: position on alignment where the starting break point was identified by RDP. Ending breakp: position on alignment where the ending break point was identified by RDP.
