# Supplementary material for: Molecular epidemiology of dengue viruses in three provinces of Lao PDR, 2006-2010
Source: PLoS Negl Trop Dis. 2018 Jan 29;12(1):e0006203. doi: 10.1371/journal.pntd.0006203 (PMC5805359; doi:10.1371/journal.pntd.0006203)
Supplement: S6 Table — The tree built with the alignment of the 3,108 DENV-1 envelope sequences downloaded from the EMBL database in April 2014 and the sequences from this study allowed the identification of 10 clusters (bootstrap >70) containing Lao strains. Genbank accession numbers of all strains from those 10 clusters are listed in this table. (DOCX) [file pntd.0006203.s007.docx]

**S6 Table. List of the DENV1 strains grouping in the ten Lao clusters.**

| Genbank accession number | Year | Country | Cluster |
| --- | --- | --- | --- |
| JQ317737 | 2008 | China | 1 |
| JQ317736 | 2008 | China | 1 |
| KY849740 | 2008 | Laos | 1 |
| KY849705 | 2008 | Laos | 1 |
| KY849704 | 2008 | Laos | 1 |
| KY849719 | 2009 | Laos | 1 |
| KY849720 | 2009 | Laos | 1 |
| KY849717 | 2009 | Laos | 1 |
| KY849715 | 2009 | Laos | 1 |
| KY849748 | 2009 | Laos | 1 |
| KY849712 | 2009 | Laos | 1 |
| KY849711 | 2009 | Laos | 1 |
| KY849714 | 2009 | Laos | 1 |
| KC182088 | 2009 | Laos | 1 |
| KY849742 | 2009 | Laos | 1 |
| KY849708 | 2009 | Laos | 1 |
| KY849716 | 2009 | Laos | 1 |
| KY849709 | 2009 | Laos | 1 |
| KY849718 | 2009 | Laos | 1 |
| KC182089 | 2009 | Laos | 1 |
| KY849701 | 2009 | Laos | 1 |
| KY849713 | 2009 | Laos | 1 |
| KY849707 | 2009 | Laos | 1 |
| KY849710 | 2009 | Laos | 1 |
| KY849723 | 2010 | Laos | 1 |
| KY849751 | 2010 | Laos | 1 |
| KY849721 | 2010 | Laos | 1 |
| KC182092 | 2010 | Laos | 1 |
| KY849722 | 2010 | Laos | 1 |
| KC182091 | 2010 | Laos | 1 |
| JN638335 | 2009 | Thailand | 1 |
| JF967878 | 2009 | Thailand | 1 |
| JF967919 | 2010 | Cambodia | 2 |
| KY849749 | 2008 | Laos | 2 |
| KC172831 | 2008 | Laos | 2 |
| KY849750 | 2009 | Laos | 2 |
| KY849727 | 2009 | Laos | 2 |
| KC182096 | 2007 | Laos | 3 |
| KC182099 | 2007 | Laos | 3 |
| JN415509 | 2007 | Laos | 3 |
| KC182098 | 2007 | Laos | 3 |
| KC182100 | 2007 | Laos | 3 |
| KC182102 | 2007 | Laos | 3 |
| KC182107 | 2008 | Laos | 3 |
| KC182105 | 2008 | Laos | 3 |
| KC182106 | 2008 | Laos | 3 |
| EU448397 | 2007 | Vietnam | 3 |
| KC172835 | 2008 | Laos | 4 |
| KC172829 | 2008 | Laos | 4 |
| KC172830 | 2008 | Laos | 4 |
| KY849745£ | 2009 | Laos | 4 |
| JQ993204 | 2007 | Thailand | 4 |
| JQ993117 | 2007 | Thailand | 4 |
| JQ993151 | 2007 | Thailand | 4 |
| JQ993149 | 2007 | Thailand | 4 |
| JQ993147 | 2007 | Thailand | 4 |
| HM181939 | 2006 | Cambodia | 5 |
| FJ639687 | 2006 | Cambodia | 5 |
| GQ868639 | 2006 | Cambodia | 5 |
| GQ868630 | 2006 | Cambodia | 5 |
| HQ624983 | 2007 | Cambodia | 5 |
| HM181945 | 2007 | Cambodia | 5 |
| HM181944 | 2007 | Cambodia | 5 |
| FJ639696 | 2007 | Cambodia | 5 |
| FJ639693 | 2007 | Cambodia | 5 |
| KF955440 | 2007 | Cambodia | 5 |
| FJ639691 | 2007 | Cambodia | 5 |
| KC182104 | 2008 | Laos | 5 |
| KF921932 | 2007 | Cambodia | 6 |
| HM631853 | 2007 | Cambodia | 6 |
| GU131919 | 2008 | Cambodia | 6 |
| GQ868635 | 2008 | Cambodia | 6 |
| GU131921 | 2008 | Cambodia | 6 |
| KY849743 | 2009 | Cambodia | 6 |
| JF967924 | 2010 | Cambodia | 6 |
| JF967928 | 2010 | Cambodia | 6 |
| KY849744 | 2009 | Laos | 6 |
| JQ403520 | 2010 | Taiwan | 6 |
| JN009085 | 2010 | China | 7 |
| JN029808 | 2010 | China | 7 |
| JQ896294 | 2010 | Ireland | 7 |
| FJ687474 | 2007 | Korea | 7 |
| KY849706 | 2008 | Laos | 7 |
| KC182103 | 2008 | Laos | 7 |
| KY849726 | 2009 | Laos | 7 |
| KY849725 | 2009 | Laos | 7 |
| KY849728 | 2009 | Laos | 7 |
| KY849724 | 2009 | Laos | 7 |
| KY849729 | 2009 | Laos | 7 |
| KY849746 | 2009 | Laos | 7 |
| KY849733 | 2010 | Laos | 7 |
| KY849736 | 2010 | Laos | 7 |
| KC182082 | 2010 | Laos | 7 |
| KC182084 | 2010 | Laos | 7 |
| KY849737 | 2010 | Laos | 7 |
| KC182109 | 2010 | Laos | 7 |
| KY849738 | 2010 | Laos | 7 |
| KC182095 | 2010 | Laos | 7 |
| KC182108 | 2010 | Laos | 7 |
| KC182111 | 2010 | Laos | 7 |
| KY849731 | 2010 | Laos | 7 |
| KY849735 | 2010 | Laos | 7 |
| KC182110 | 2010 | Laos | 7 |
| KY849734 | 2010 | Laos | 7 |
| KC182112 | 2010 | Laos | 7 |
| KY849732 | 2010 | Laos | 7 |
| KY849739 | 2010 | Laos | 7 |
| KY849730 | 2010 | Laos | 7 |
| KY849703 | 2010 | Laos | 7 |
| KY849702 | 2010 | Laos | 7 |
| KY849747 | 2010 | Laos | 7 |
| KF926700 | 2011 | Laos | 7 |
| JF967921 | 2010 | Thailand | 7 |
| JF967940 | 2010 | Thailand | 7 |
| EF508204 | 2006 | China | 8 |
| JQ317743 | 2006 | China | 8 |
| JQ317744 | 2006 | China | 8 |
| JQ317745 | 2006 | China | 8 |
| KJ415097 | 2013 | China | 8 |
| KC182085 | 2008 | Laos | 8 |
| JF960217 | 2009 | Singapore | 8 |
| JF960219 | 2009 | Singapore | 8 |
| JF960220 | 2010 | Singapore | 8 |
| HQ891313 | 2009 | Sri Lanka | 8 |
| HQ891315 | 2009 | Sri Lanka | 8 |
| HQ891314 | 2009 | Sri Lanka | 8 |
| JN054256 | 2009 | Sri Lanka | 8 |
| HQ891316 | 2009 | Sri Lanka | 8 |
| JN054255 | 2010 | Sri Lanka | 8 |
| JN638327 | 2009 | Thailand | 8 |
| KC182101 | 2007 | Laos | 9 |
| JQ403517 | 2008 | Taiwan | 9 |
| AB608787 | 2008 | Taiwan | 9 |
| JQ993193 | 2006 | Thailand | 9 |
| JQ993148 | 2007 | Thailand | 9 |
| JQ993150 | 2007 | Thailand | 9 |
| EU448394 | 2007 | Thailand | 9 |
| KC172832 | 2009 | Laos | 10 |
| JQ993122 | 2006 | Thailand | 10 |
| JQ993195 | 2006 | Thailand | 10 |
| JQ993156 | 2006 | Thailand | 10 |
| JQ993155 | 2006 | Thailand | 10 |
| JQ993153 | 2006 | Thailand | 10 |
| JQ993121 | 2006 | Thailand | 10 |
| JQ993154 | 2006 | Thailand | 10 |
| JQ993187 | 2006 | Thailand | 10 |
| JQ993125 | 2006 | Thailand | 10 |
| JQ993170 | 2006 | Thailand | 10 |
| JQ993175 | 2006 | Thailand | 10 |
| JQ993166 | 2006 | Thailand | 10 |
| JQ993159 | 2006 | Thailand | 10 |
| JQ993190 | 2006 | Thailand | 10 |
| JQ993197 | 2006 | Thailand | 10 |
| JQ993163 | 2006 | Thailand | 10 |
| JQ993152 | 2006 | Thailand | 10 |
| JQ993179 | 2006 | Thailand | 10 |
| JQ993161 | 2006 | Thailand | 10 |
| JQ993126 | 2006 | Thailand | 10 |
| JQ993162 | 2006 | Thailand | 10 |
| JQ993124 | 2006 | Thailand | 10 |
| JQ993164 | 2006 | Thailand | 10 |
| JQ993128 | 2006 | Thailand | 10 |
| JQ993123 | 2006 | Thailand | 10 |
| JQ993120 | 2006 | Thailand | 10 |
| JQ993119 | 2006 | Thailand | 10 |
| JQ993158 | 2006 | Thailand | 10 |
| JQ993181 | 2006 | Thailand | 10 |
| JQ993129 | 2006 | Thailand | 10 |
| JQ993186 | 2006 | Thailand | 10 |
| JQ993118 | 2006 | Thailand | 10 |
| JQ993160 | 2006 | Thailand | 10 |
| JQ993169 | 2006 | Thailand | 10 |
| JQ993172 | 2006 | Thailand | 10 |
| JQ993168 | 2006 | Thailand | 10 |
| JQ993157 | 2006 | Thailand | 10 |
| JQ993185 | 2006 | Thailand | 10 |
| JQ993182 | 2006 | Thailand | 10 |
| JQ993165 | 2006 | Thailand | 10 |
| JQ993176 | 2006 | Thailand | 10 |
| JQ993180 | 2006 | Thailand | 10 |
| JQ993138 | 2007 | Thailand | 10 |
| JQ993142 | 2007 | Thailand | 10 |
| JQ993113 | 2007 | Thailand | 10 |
| JQ993116 | 2007 | Thailand | 10 |
| JQ993136 | 2007 | Thailand | 10 |
| JQ993202 | 2007 | Thailand | 10 |
| JQ993144 | 2007 | Thailand | 10 |
| JQ993145 | 2007 | Thailand | 10 |
| JQ993114 | 2007 | Thailand | 10 |
| JQ993115 | 2007 | Thailand | 10 |
| JQ993109 | 2007 | Thailand | 10 |
| HM469967 | 2007 | Thailand | 10 |
| JQ993137 | 2007 | Thailand | 10 |
| JQ993146 | 2007 | Thailand | 10 |
| JQ993140 | 2007 | Thailand | 10 |
| JN415526 | 2008 | Thailand | 10 |
| JF967812 | 2008 | Thailand | 10 |
| JF967862 | 2009 | Thailand | 10 |
| HG316481 | 2010 | Thailand | 10 |
