# Supplementary figures and images for: Molecular epidemiology of dengue viruses in three provinces of Lao PDR, 2006-2010
Source: PLoS Negl Trop Dis. 2018 Jan 29;12(1):e0006203. doi: 10.1371/journal.pntd.0006203 (PMC5805359; doi:10.1371/journal.pntd.0006203)

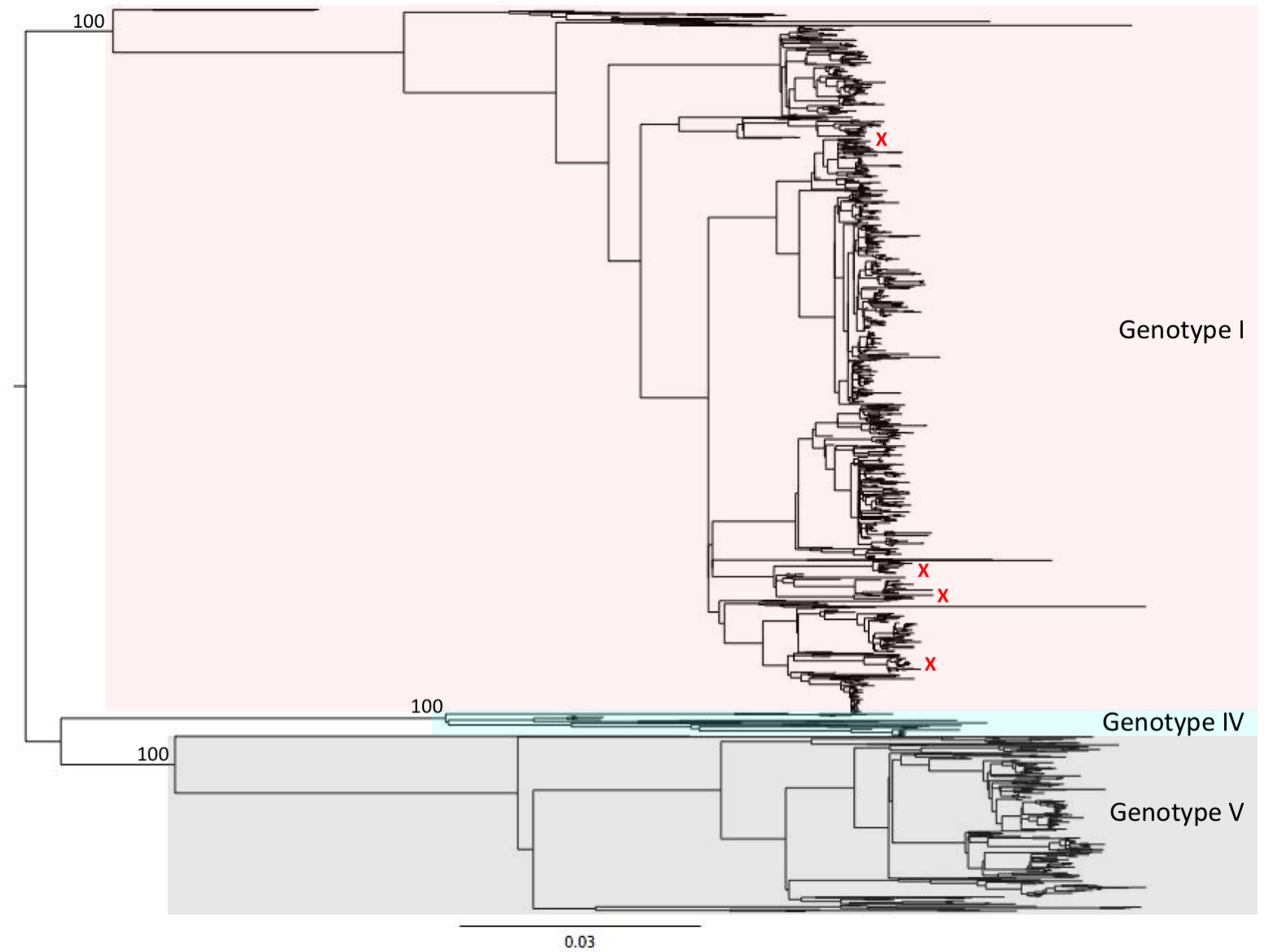

Supplement: S1 Fig — 1,373 DENV-1 complete CDS downloaded from the EMBL database in April 2014 were aligned with the DENV-1 sequences from this study. The tree was built using the maximum-likelihood (ML) method (General Time Reversible Model with invariant sites and a gamma distribution of rates across sites) and bootstrap resampling with 500 replicates on the RAXML8.0.0 software. Bootstrap values are only displayed for the genotype nodes. Genotypes distribution is illustrated by color-shaded boxes. Red crosses indicate the location of the strains sequenced in this study. (TIFF) [file pntd.0006203.s008.tiff]

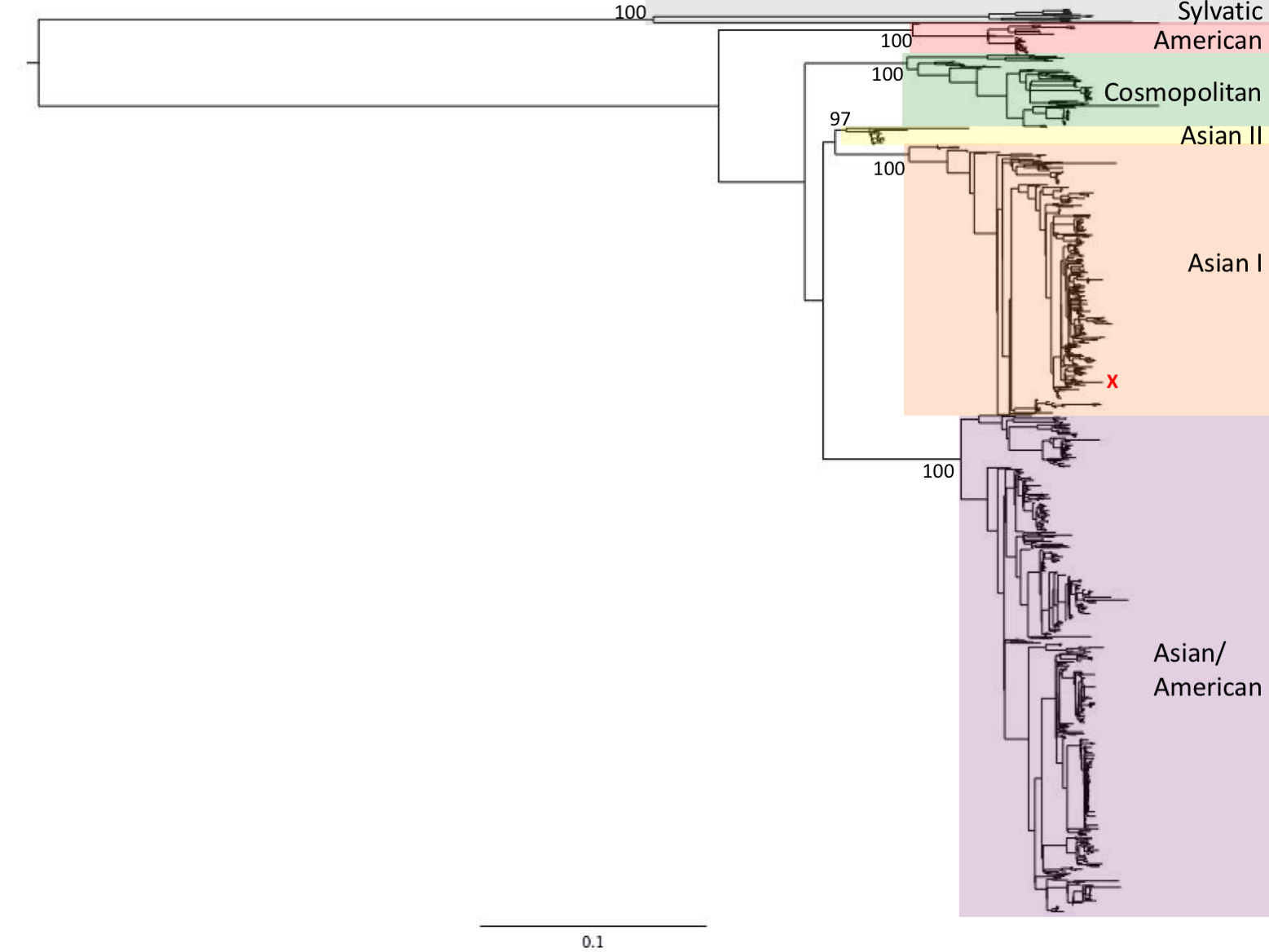

Supplement: S2 Fig — 998 DENV-2 complete CDS downloaded from the EMBL database in April 2014 were aligned with the DENV-2 sequences from this study. The tree was built using a maximum-likelihood (ML) method (General Time-Reversible Model with gamma distributed rates across sites) and bootstrap resampling with 500 replicates on the RAXML8.0.0 software. Bootstrap values are only displayed for the genotype nodes. Genotypes (Sylvatic, American, Cosmopolitan, Asian I and II, Asian/American) distribution is illustrated by color-shaded boxes. Red crosses indicate the location of the strains sequenced in this study. (TIFF) [file pntd.0006203.s009.tiff]

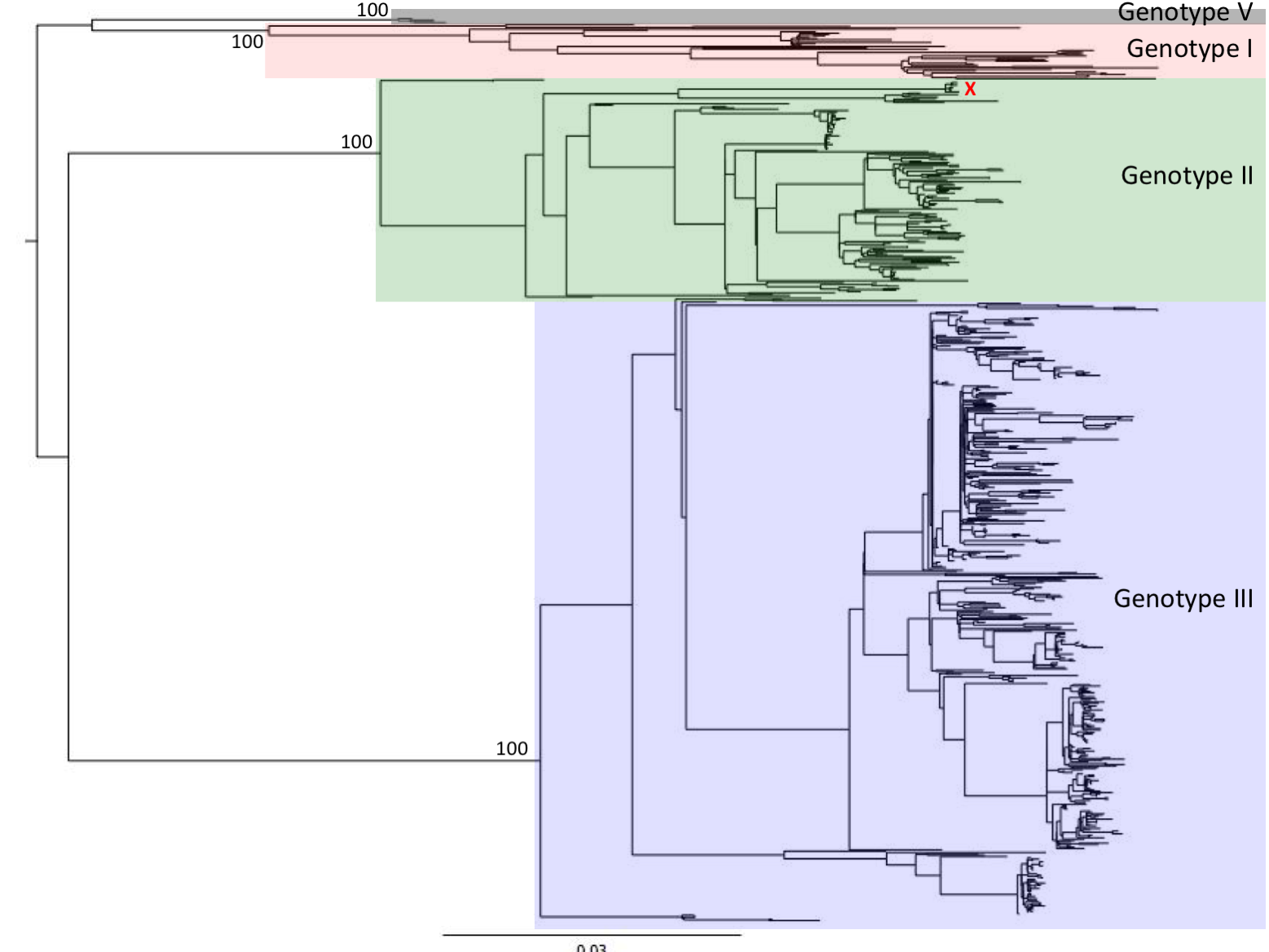

Supplement: S3 Fig — 670 DENV-3 complete CDS downloaded from the EMBL database in April 2014 were aligned with the DENV-3 sequences from this study. The tree was built using a maximum-likelihood (ML) method (Tamura-Nei model with gamma distributed rates across sites) and bootstrap resampling with 500 replicates on the RAXML8.0.0 software. Bootstrap values are only displayed for the genotype nodes. Genotypes distribution is illustrated color-shaded boxes. Red crosses indicate the location of the strains sequenced in this study. (TIFF) [file pntd.0006203.s010.tiff]

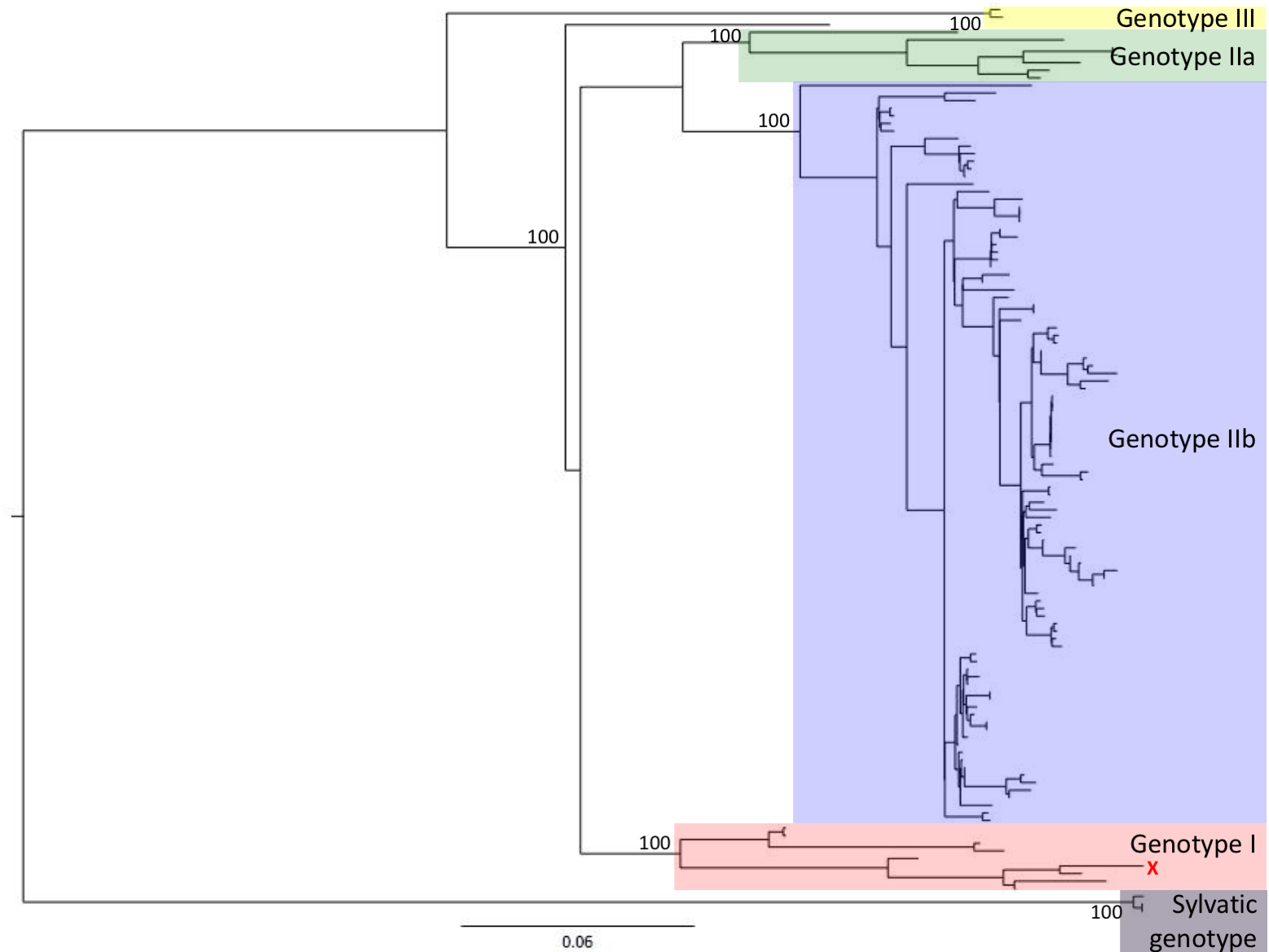

Supplement: S4 Fig — 123 DENV-4 complete CDS downloaded from the EMBL database in April 2014 were aligned with the DENV-4 sequences from this study. The tree was built using a maximum-likelihood (ML) method (General Time Reversible Model with invariant sites and a gamma distribution of rates across sites) and bootstrap resampling with 500 replicates on the RAXML8.0.0 software. Bootstrap values are only displayed for the genotype nodes. Genotypes distribution is illustrated by color-shaded boxes. Red crosses indicate the location of the strains sequenced in this study. (TIFF) [file pntd.0006203.s011.tiff]

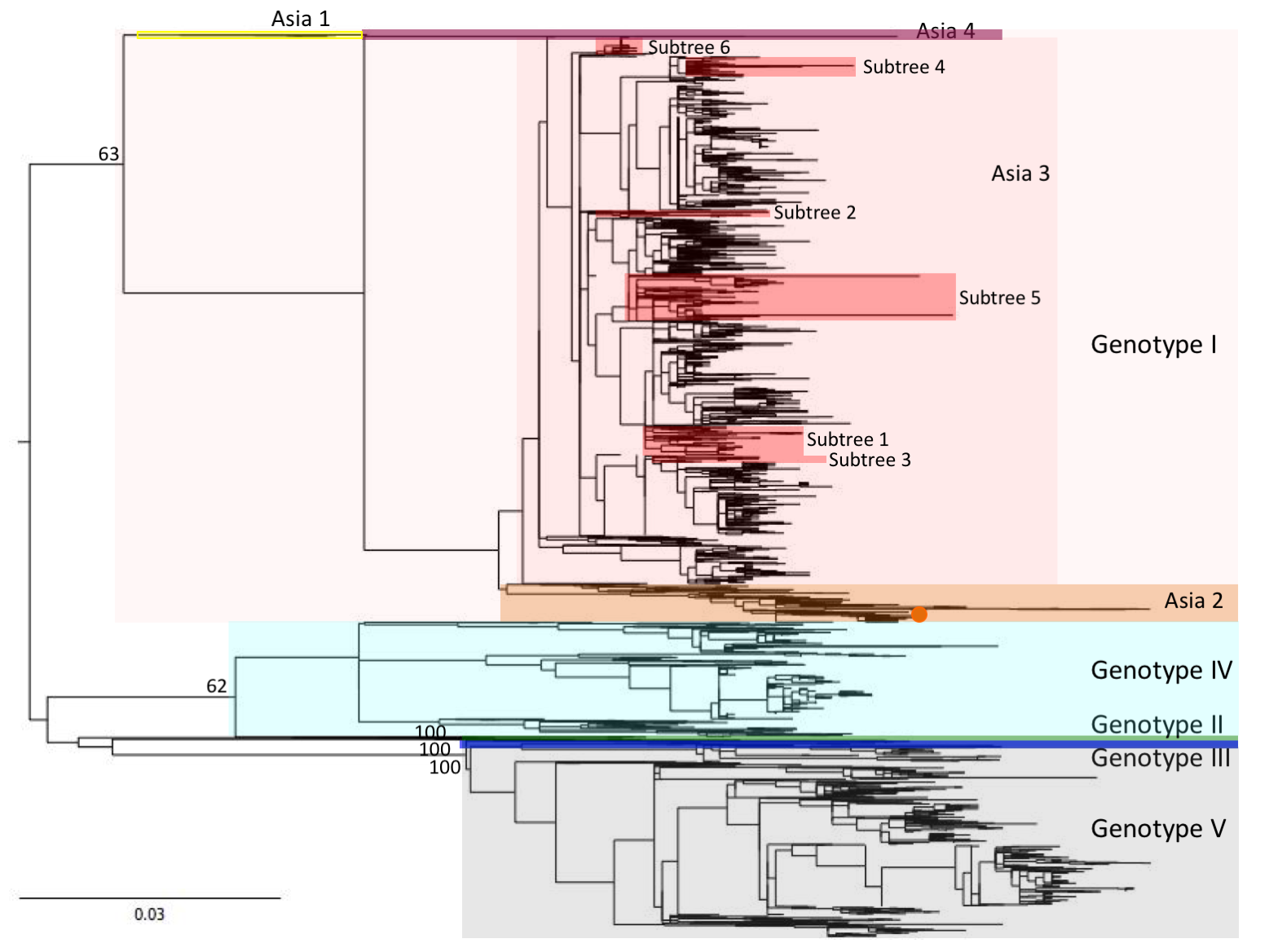

Supplement: S5 Fig — 3,108 DENV-1 envelope sequences downloaded from the EMBL database in April 2014 were aligned with the DENV-1 sequences from this study. The tree was built using a maximum-likelihood (ML) method (General Time Reversible Model with invariant sites and a gamma distribution of rates across sites) and bootstrap resampling with 500 replicates on the RAXML8.0.0 software. Bootstrap values are only displayed for the genotype nodes. Genotypes distribution and clades Asia 1 to 4 within Genotype I are illustrated by color-shaded boxes. Six subtrees containing Lao strains were extracted from this tree and displayed in more details in S9 Fig. The orange circle indicates the location of the 1996 Lao strain. (TIFF) [file pntd.0006203.s012.tiff]

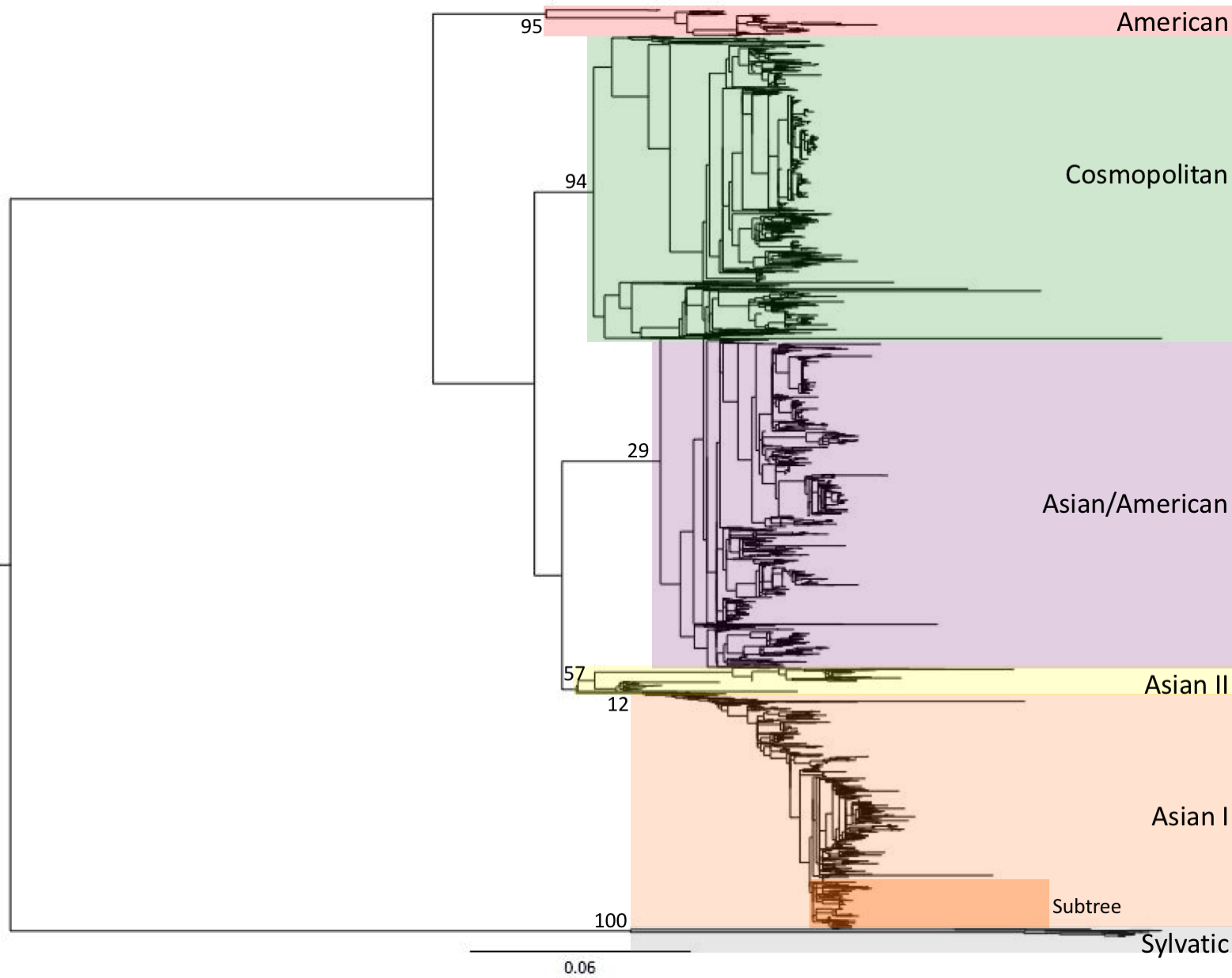

Supplement: S6 Fig — 3,067 DENV-2 envelop sequences downloaded from the EMBL database in April 2014 were aligned with the DENV-2 sequences from this study. The tree was built using a maximum-likelihood (ML) method (General Time-Reversible Model with gamma distributed rates across sites) and bootstrap resampling with 500 replicates on the RAXML8.0.0 software. Bootstrap values are only displayed for the genotype nodes. Genotypes (Sylvatic, American, Cosmopolitan, Asian I and II, Asian/American) distribution is illustrated by color-shaded boxes. A subtree containing Lao strains was extracted from this tree and displayed in details in S10 Fig. (TIFF) [file pntd.0006203.s013.tiff]

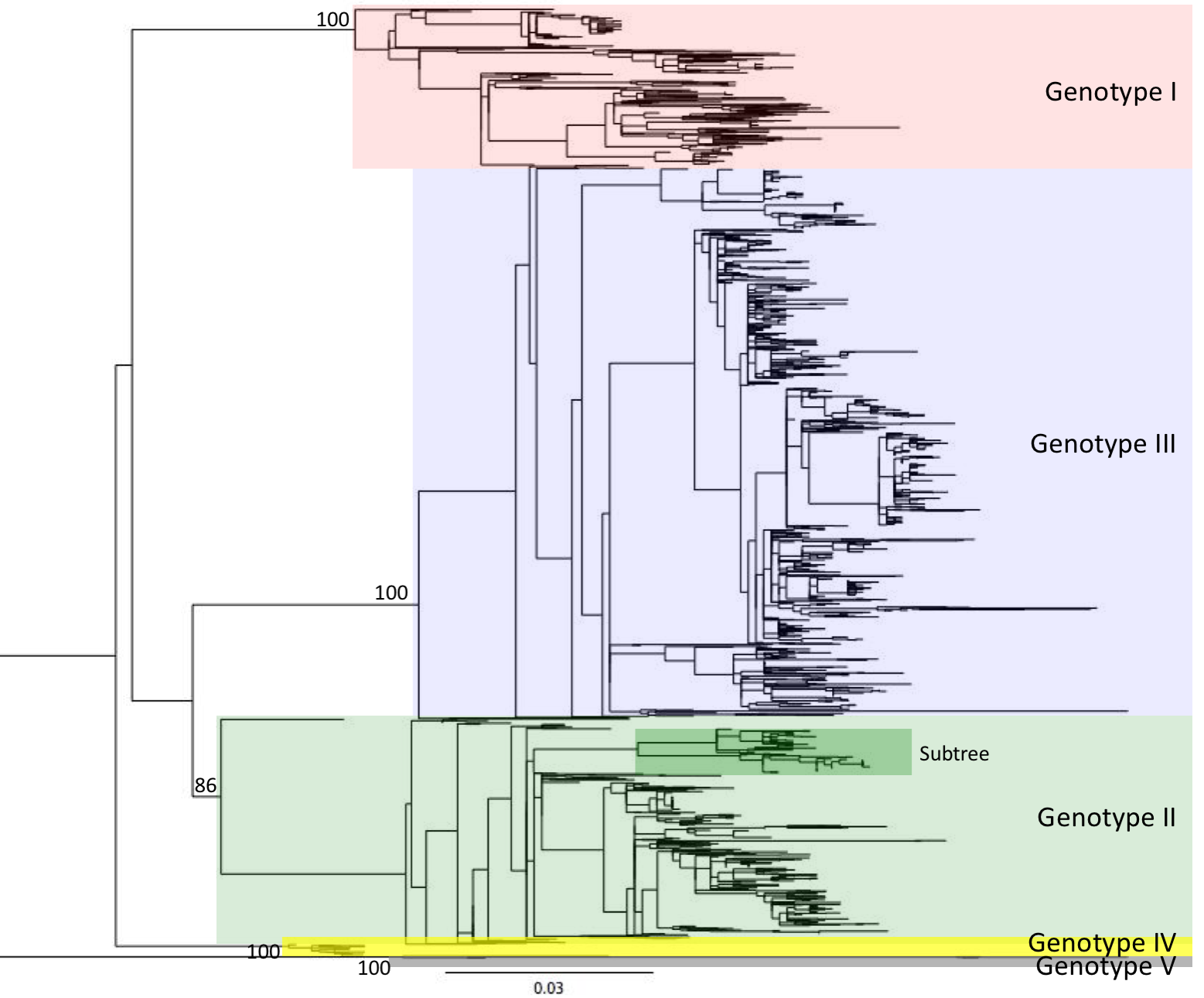

Supplement: S7 Fig — 1,817 DENV-3 envelop sequences downloaded from the EMBL database in April 2014 were aligned with the DENV-3 sequences from this study. The tree was built using a maximum-likelihood (ML) method (Tamura-Nei model with gamma distributed rates across sites) and bootstrap resampling with 500 replicates on the RAXML8.0.0 software. Bootstrap values are only displayed for the genotype nodes. Genotypes distribution is illustrated by color-shaded boxes. A subtree containing Lao strains was extracted from this tree and displayed in details in S11 Fig. (TIFF) [file pntd.0006203.s014.tiff]

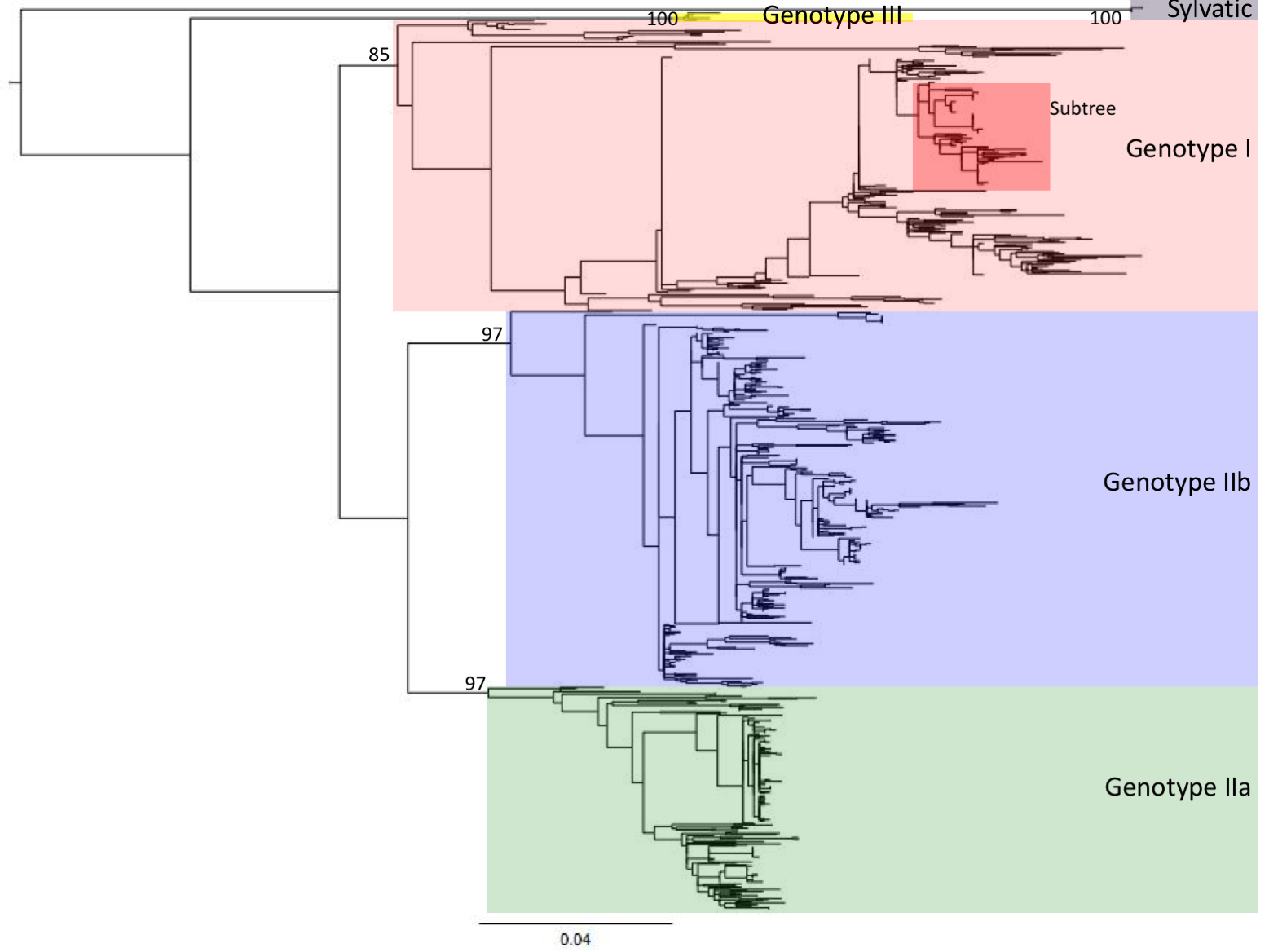

Supplement: S8 Fig — 859 DENV-4 envelop sequences downloaded from the EMBL database in April 2014 were aligned with the DENV-4 sequences from this study. The tree was built using a maximum-likelihood (ML) method (General Time Reversible Model with invariant sites and a gamma distribution of rates across sites) and bootstrap resampling with 500 replicates on the RAXML8.0.0 software. Bootstrap values are only displayed for the genotype nodes. Genotypes distribution is illustrated by color-shaded boxes. A subtree containing Lao strains was extracted from this tree and displayed in details in S12 Fig. (TIFF) [file pntd.0006203.s015.tiff]

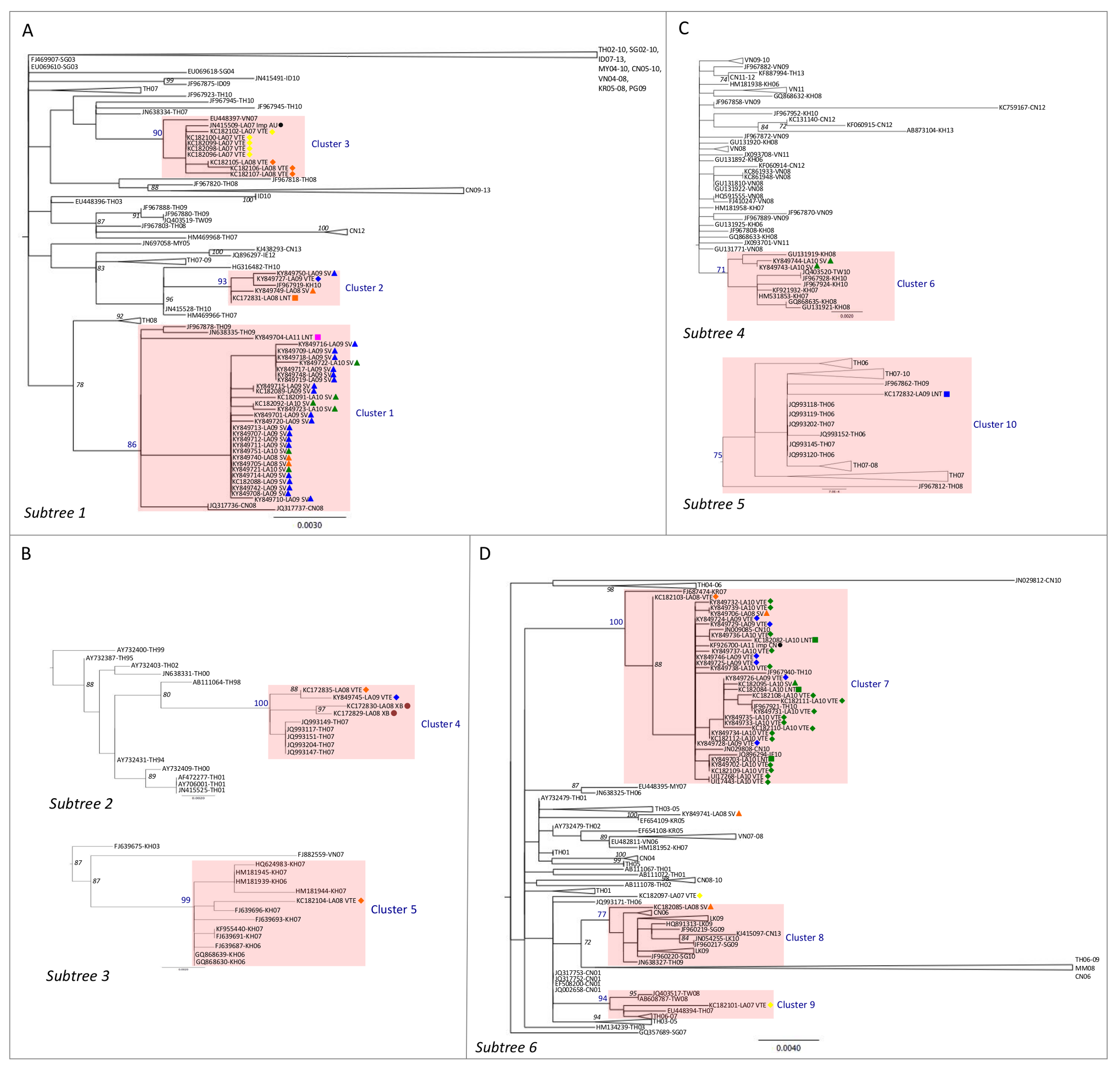

Supplement: S9 Fig — Six subtrees containing Lao strains were extracted from the tree built with the complete data set of DENV-1 envelop sequences displayed in S5 Fig. Only bootstrap values over 70 are reported on the figure. The Lao strains are dispersed in ten clusters (bootstrat>70): clusters 1, 2 and 3 in subtree 1 (A); cluster 4 and 5 in subtrees 2 and 3 respectively (B); clusters 6 and 10 in subtrees 4 and 5 respectively (C); clusters 7, 8 and 9 in subtree 6 (D). Strains from Vientiane are indicated by diamonds, strains from LNT by squares and strains from SV by triangles. Strains isolated in 2007 are in yellow, 2008 in orange, 2009 in blue, 2010 in green, and 2011 in pink. Other Lao strains are indicated by brown circles. Lao strains from imported cases are indicated by black circles. Strains are indicated by Genbank accession number followed by country (ISO code) and year of isolation. For the nodes that have been collapsed for simplification purposes (shown by triangles), descendant strains are only described by country and years. The province of origin is indicated for the strains from this study (VTE: Vientiane, SV: Salavan, LNT: Luang Namtha). (TIF) [file pntd.0006203.s016.tif]

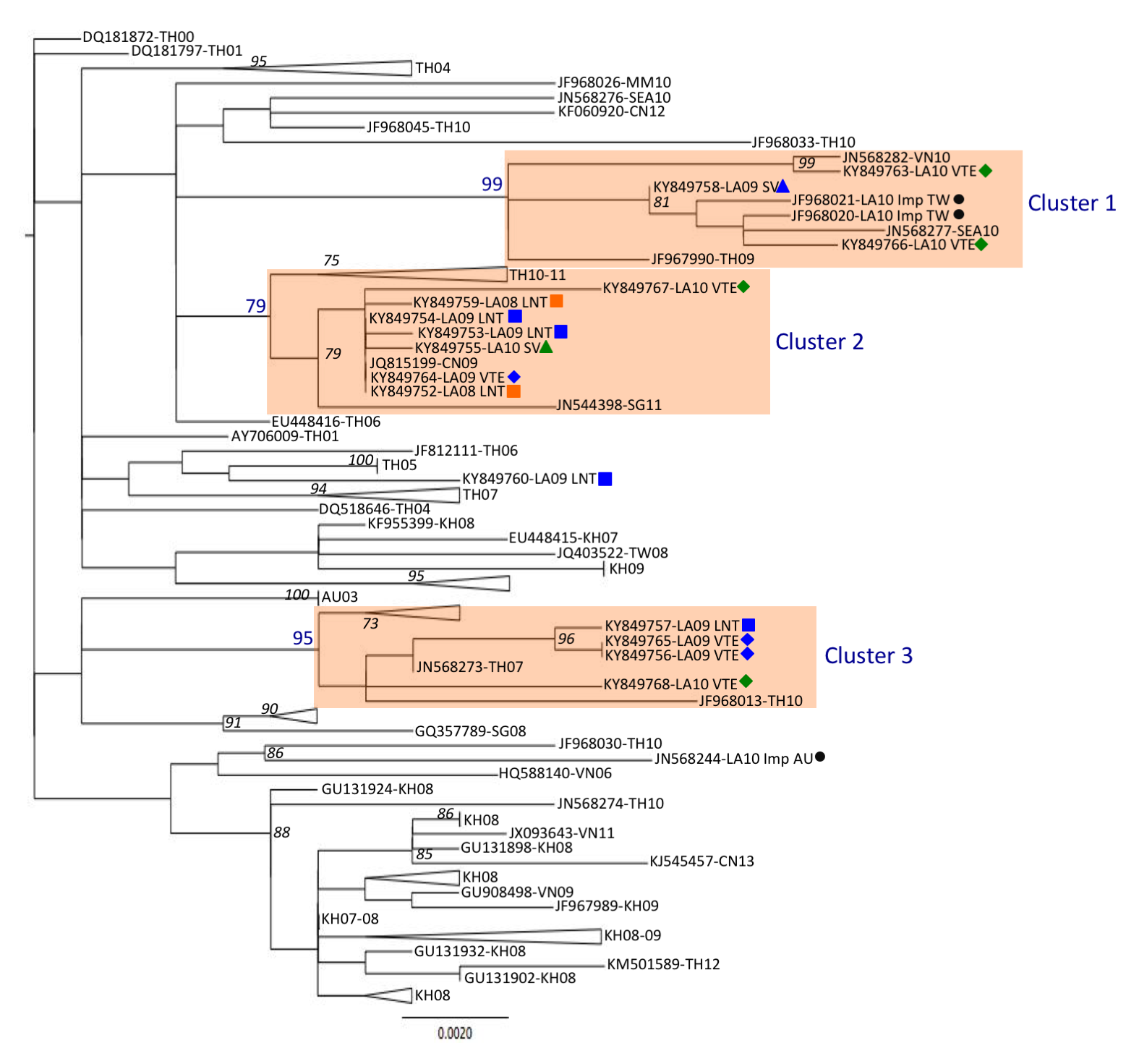

Supplement: S10 Fig — A subtree containing Lao strains was extracted from the tree built with the complete data set of DENV-2 envelop sequences displayed in S6 Fig. Only bootstrap values over 70 are reported on the figure. The Lao strains are dispersed in three clusters (bootstrat>70). Strains from Vientiane are indicated by diamonds, strains from LNT by squares and strains from SV by triangles. Strains isolated in 2008 are in orange, 2009 in blue, and 2010 in green. Lao strains from imported cases are shown by black circles. Genbank accession numbers followed by country (ISO code) and year of isolation are provided for each strain. For the nodes that have been collapsed for simplification purposes (shown by triangles), descendant strains are only described by country and years. The province of origin is indicated for the strains from this study (VTE: Vientiane, SV: Salavan, LNT: Luang Namtha). (TIFF) [file pntd.0006203.s017.tiff]

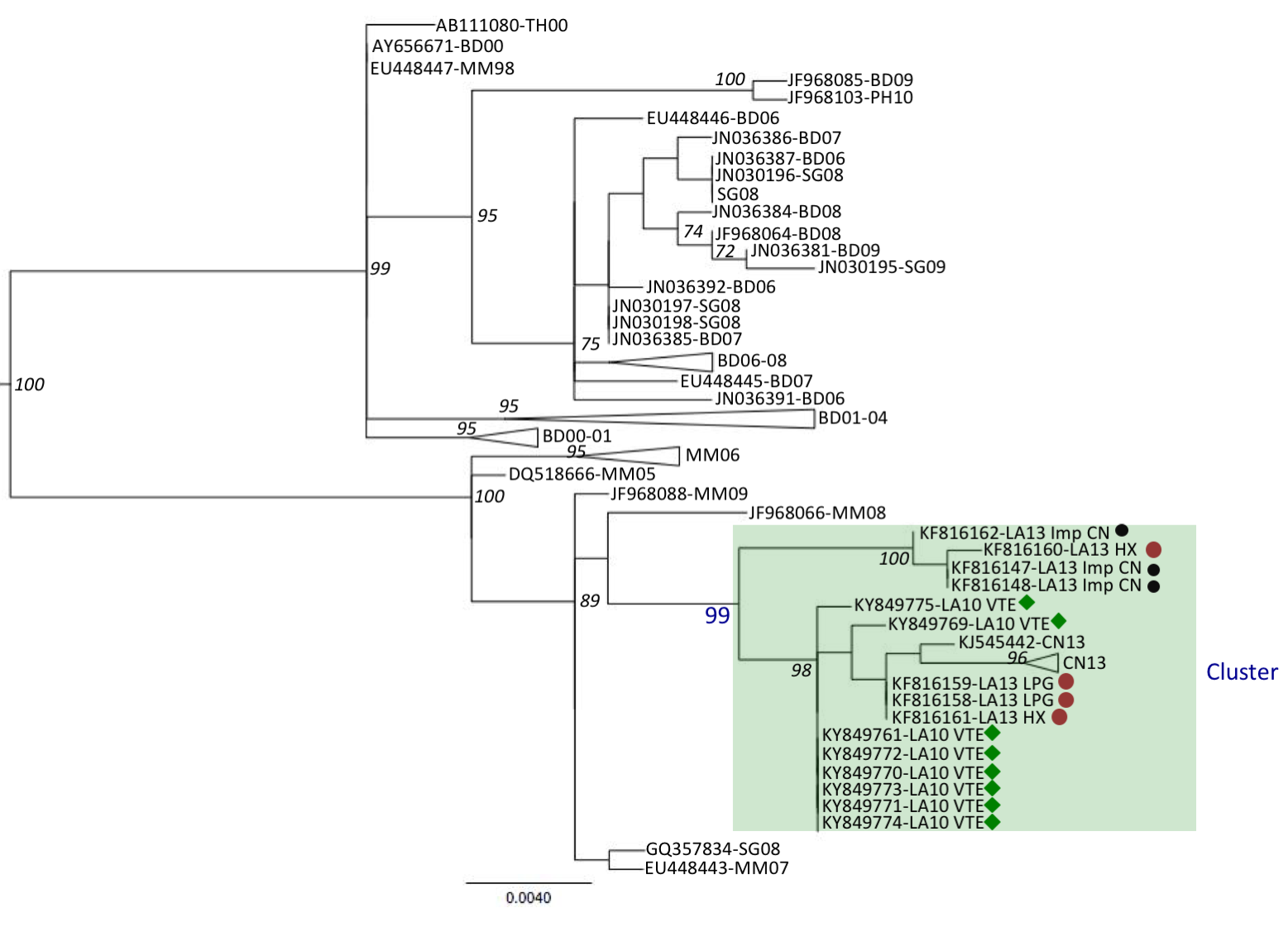

Supplement: S11 Fig — A subtree containing Lao strains was extracted from the tree built with the complete data set of DENV-3 envelop sequences displayed in S7 Fig. Only bootstrap values over 70 are reported on the figure. The Lao strains are grouped in one cluster (bootstrat = 99). All the strains from this study were from 2010 and from Vientiane. They are displayed by green diamonds. Other Lao strains are shown by brown circles. Lao strains from imported cases are indicated by black circles. Genbank accession numbers, followed by country (ISO code) and year of isolation are provided for each strain. For the nodes that have been collapsed for simplification purposes (shown by triangles), descendant strains are only described by country and years. The province of origin is indicated for the strains from this study (VTE: Vientiane). (TIFF) [file pntd.0006203.s018.tiff]

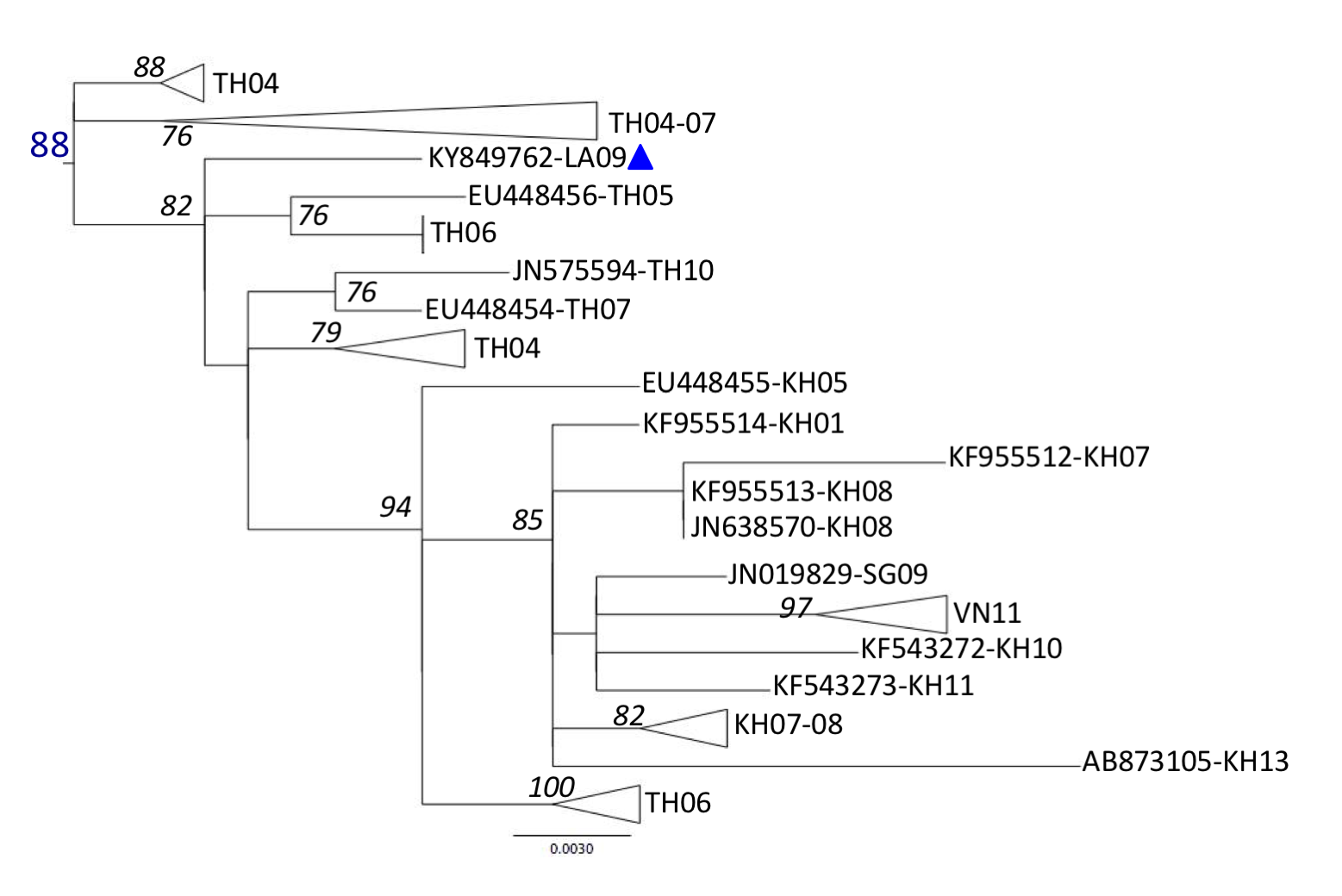

Supplement: S12 Fig — A subtree containing the Lao strain was extracted from the tree built with the complete data set of DENV-4 envelope sequences displayed in S8 Fig. The Lao strain from this study was from Salavan from 2009 and is shown by a blue triangle. Genbank accession numbers followed by country (ISO code) and year of isolation are provided for each strain. For the nodes that have been collapsed for simplification purposes (shown by triangles), descendant strains are only described by country and years. The province of origin is indicated for the strain from this study (SV: Salavan). (TIFF) [file pntd.0006203.s019.tiff]
